# *In situ* resource utilization of lunar soil for highly efficient extraterrestrial fuel and oxygen supply

**DOI:** 10.1093/nsr/nwac200

**Published:** 2022-09-23

**Authors:** Yuan Zhong, Jingxiang Low, Qing Zhu, Yawen Jiang, Xiwen Yu, Xinyu Wang, Fei Zhang, Weiwei Shang, Ran Long, Yingfang Yao, Wei Yao, Jun Jiang, Yi Luo, Weihua Wang, Jinlong Yang, Zhigang Zou, Yujie Xiong

**Affiliations:** Hefei National Research Center for Physical Sciences at the Microscale, School of Chemistry and Materials Science, National Synchrotron Radiation Laboratory, School of Information Science and Technology, University of Science and Technology of China, Hefei 230026, China; Hefei National Research Center for Physical Sciences at the Microscale, School of Chemistry and Materials Science, National Synchrotron Radiation Laboratory, School of Information Science and Technology, University of Science and Technology of China, Hefei 230026, China; Hefei National Research Center for Physical Sciences at the Microscale, School of Chemistry and Materials Science, National Synchrotron Radiation Laboratory, School of Information Science and Technology, University of Science and Technology of China, Hefei 230026, China; Hefei National Research Center for Physical Sciences at the Microscale, School of Chemistry and Materials Science, National Synchrotron Radiation Laboratory, School of Information Science and Technology, University of Science and Technology of China, Hefei 230026, China; Eco-Materials and Renewable Energy Research Center (ERERC), Jiangsu Key Laboratory for Nano Technology, National Laboratory of Solid State Microstructures, School of Physics, Collaborative Innovation Center of Advanced Microstructures, College of Engineering and Applied Sciences, Nanjing University, Nanjing 210093, China; Hefei National Research Center for Physical Sciences at the Microscale, School of Chemistry and Materials Science, National Synchrotron Radiation Laboratory, School of Information Science and Technology, University of Science and Technology of China, Hefei 230026, China; Hefei National Research Center for Physical Sciences at the Microscale, School of Chemistry and Materials Science, National Synchrotron Radiation Laboratory, School of Information Science and Technology, University of Science and Technology of China, Hefei 230026, China; Hefei National Research Center for Physical Sciences at the Microscale, School of Chemistry and Materials Science, National Synchrotron Radiation Laboratory, School of Information Science and Technology, University of Science and Technology of China, Hefei 230026, China; Hefei National Research Center for Physical Sciences at the Microscale, School of Chemistry and Materials Science, National Synchrotron Radiation Laboratory, School of Information Science and Technology, University of Science and Technology of China, Hefei 230026, China; Eco-Materials and Renewable Energy Research Center (ERERC), Jiangsu Key Laboratory for Nano Technology, National Laboratory of Solid State Microstructures, School of Physics, Collaborative Innovation Center of Advanced Microstructures, College of Engineering and Applied Sciences, Nanjing University, Nanjing 210093, China; Qian Xuesen Laboratory of Space Technology, China Academy of Space Technology, Beijing 100094, China; Hefei National Research Center for Physical Sciences at the Microscale, School of Chemistry and Materials Science, National Synchrotron Radiation Laboratory, School of Information Science and Technology, University of Science and Technology of China, Hefei 230026, China; Hefei National Research Center for Physical Sciences at the Microscale, School of Chemistry and Materials Science, National Synchrotron Radiation Laboratory, School of Information Science and Technology, University of Science and Technology of China, Hefei 230026, China; Qian Xuesen Laboratory of Space Technology, China Academy of Space Technology, Beijing 100094, China; Hefei National Research Center for Physical Sciences at the Microscale, School of Chemistry and Materials Science, National Synchrotron Radiation Laboratory, School of Information Science and Technology, University of Science and Technology of China, Hefei 230026, China; Eco-Materials and Renewable Energy Research Center (ERERC), Jiangsu Key Laboratory for Nano Technology, National Laboratory of Solid State Microstructures, School of Physics, Collaborative Innovation Center of Advanced Microstructures, College of Engineering and Applied Sciences, Nanjing University, Nanjing 210093, China; Hefei National Research Center for Physical Sciences at the Microscale, School of Chemistry and Materials Science, National Synchrotron Radiation Laboratory, School of Information Science and Technology, University of Science and Technology of China, Hefei 230026, China

**Keywords:** Chang’E-5 lunar soil, fuel production, CO_2_ conversion, extraterrestrial settlement, *in situ* resource utilization

## Abstract

Building up a lunar settlement is the ultimate aim of lunar exploitation. Yet, limited fuel and oxygen supplies restrict human survival on the Moon. Herein, we demonstrate the *in situ* resource utilization of lunar soil for extraterrestrial fuel and oxygen production, which may power up our solely natural satellite and supply respiratory gas. Specifically, the lunar soil is loaded with Cu species and employed for electrocatalytic CO_2_ conversion, demonstrating significant production of methane. In addition, the selected component in lunar soil (i.e. MgSiO_3_) loaded with Cu can reach a CH_4_ Faradaic efficiency of 72.05% with a CH_4_ production rate of 0.8 mL/min at 600 mA/cm^2^. Simultaneously, an O_2_ production rate of 2.3 mL/min can be achieved. Furthermore, we demonstrate that our developed process starting from catalyst preparation to electrocatalytic CO_2_ conversion is so accessible that it can be operated in an unmmaned manner via a robotic system. Such a highly efficient extraterrestrial fuel and oxygen production system is expected to push forward the development of mankind's civilization toward an extraterrestrial settlement.

## Teaser

This work demonstrates the *in situ* resource utilization of Chang’E-5 lunar soil for electrocatalytic CO_2_ conversion toward fuel and oxygen production in an unmanned operation manner via the robotic system.

## INTRODUCTION

To achieve rational extraterrestrial exploitation and settlement on the Moon, the sustainable supply of fuels and oxygen is an indispensable issue [1,2]. The artificial production of hydrocarbon fuels (e.g. methane (CH_4_) and ethylene (C_2_H_4_)) along with oxygen using carbon dioxide (CO_2_) and water (H_2_O) as the feedstocks via a combination of photovoltaic and electrocatalysis is demonstrably feasible on Earth [3–5] and has been known as a potential strategy to be imitated at extraterrestrial sites. With the advancement in lunar exploration, it has been discovered that the lunar surface possesses CO_2_ and H_2_O reserves [6,7], further supporting this proposal. For example, Schorghofer *et al*. discovered the presence of carbon dioxide cold traps on the Moon [8], which could provide a sufficient carbon source for human activity. In this regard, such a strategy is one of the most likely fuel and oxygen production technologies to be first implemented on the Moon. Yet, it appears that electrocatalytic CO_2_ conversion can only be operated on a laboratory scale at extraterrestrial sites, which is an order of magnitude too small to accommodate the human energy requirements, due to the lack of efficient catalysts, stable electrolyser architectures, etc. Among them, catalysts play the dominant role in determining the efficiency and stability of electrocatalytic reactions. Therefore, this strategy can arguably be achieved with the exploration of appropriate catalysts from the Moon [9].

Recently, *in situ* resource utilization (ISRU) technology, which aims to enhance extraterrestrial exploration efficiency and reduce the amount of the resources that have to be transported from the Earth during extraterrestrial missions [10], has attracted wide attention from the scientific community for overcoming the limited transportation load of a spacecraft [11]. Such technology requires an ample understanding of the composition of the target extraterrestrial sites [10]. Up to the present, humans have performed 10 lunar sample return missions, including 6 Apollo, 3 Luna and 1 Chang’E (CE) mission [12], which enrich our knowledge of the composition of lunar regolith [13,14], endowing wide opportunities for expediting the development of ISRU. Typically, the composition of lunar regolith is rather simple with augite, plagioclase, olivine and ilmenite as the four main minerals in the returned lunar soils [15]. As such, it is conceivable that the effective use of these components for electrocatalyst production can greatly facilitate the exploitation of electrocatalytic fuel production on the Moon for sustainably supplying fuels.

Over and above that, labor force is valuable and limited at extraterrestrial sites. For this reason, alternatives to manned operation are highly sought after and the utilization of robotic systems for electrocatalytic CO_2_ conversion can be a potential answer [16]. This solution raises a more stringent requirement for electrocatalytic system, whose entire unmanned process starting from catalyst preparation to electrocatalytic CO_2_ conversion should be readily accessible. Herein, we first demonstrate the feasibility of ISRU of lunar soil obtained by the CE-5 return mission for electrocatalytic CO_2_ conversion toward hydrocarbon fuel and oxygen production. We then analyse the active components in the lunar soil for the electrocatalytic reaction, establishing a highly accessible catalyst preparation procedure. To further show the high practicability of such a system on the Moon, we employ a robotic system to perform the unmanned catalyst preparation and electrolyser assembly toward electrocatalytic CO_2_ conversion. Our work represents an important strategy for sustainably supplying fuels and oxygen toward reaching a human settlement on the Moon.

## RESULTS

### Electrocatalytic CO_2_ conversion over lunar soil

**Figure 1.**
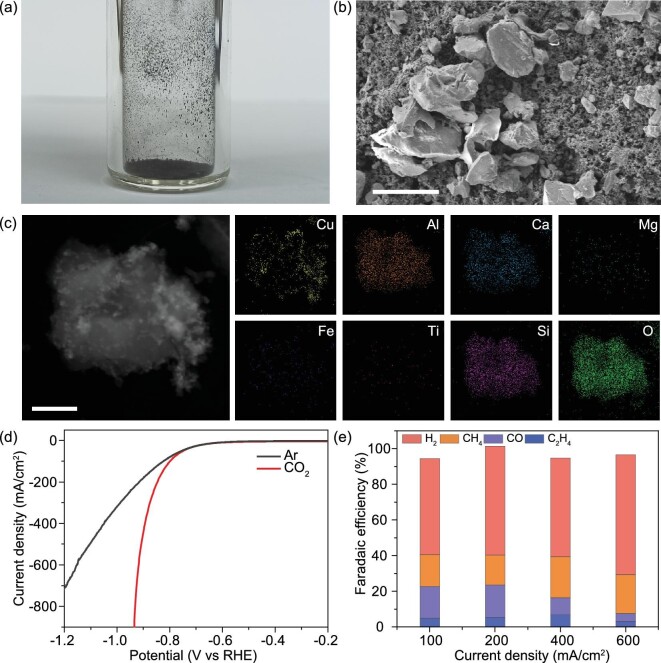
Physicochemical properties of the lunar soil (CE-5 sample). (a) Optical image of lunar soil. (b) Scanning electron microscope (SEM) image of the Cu/lunar soil. Scale bar: 20 μm. (c) Elemental mapping images of the Cu/lunar soil for Cu, Al, Ca, Mg, Fe, Ti, Si and O elements. Scale bar: 200 nm. (d) LSV curves of Cu/lunar soil with different feeding gases. (e) Faradaic efficiency for H_2_, CH_4_, CO and C_2_H_4_ of Cu/lunar soil in electrocatalytic CO_2_ conversion at different current densities.

In this work, we have obtained lunar soil from the CE-5 return mission (see Fig. 1a), the first sample of lunar regolith brought back to Earth since the Luna 24 mission in 1976 [17]. Given that we wish to employ this lunar soil for electrocatalytic CO_2_ conversion, we first modify the lunar soil with the Cu species, which have been proven to be perfect active sites for CO_2_ conversion [18,19]. As shown in Fig. 1b, the lunar soil demonstrates a bulk structure. The elemental mapping images (Fig. 1c) indicate that, apart from the Cu element, Al, Ca, Mg, Fe, Ti, Si and O elements can be determined on the Cu-loaded lunar soil (Cu/lunar soil). These elements are in good accordance with the composition of the most common pyroxene—augite [20], which is a silicate of calcium, magnesium, iron, titanium and aluminum. Upon Cu modification, we perform the electrocatalytic CO_2_ conversion test in the flow cell (see Supplementary Fig. S1) with a gas diffusion electrode to overcome the CO_2_ solubility limit in the aqueous solution. We begin the test by obtaining the linear sweep voltammetry (LSV) curves of the prepared samples in a flow cell with Ar and CO_2_ feeding gases (Fig. 1d). The result shows that the current density in the flow cell with CO_2_ feeding gas is significantly enhanced in low input potential compared to that with Ar feeding gas, suggesting the high tendency of the Cu/lunar soil toward CO_2_ conversion. In addition, it has been discovered that the product selectivity of the Cu/lunar soil can be easily tuned by changing the Cu content and all the products produced through electrocatalytic CO_2_ conversion using Cu/lunar soil are valuable fuels (Fig. 1e)and Supplementary Fig. S2), i.e. H_2_, CH_4_, CO and C_2_H_4_, confirming the high feasibility of lunar soil being employed for fuel production. Notably, all these products are gaseous fuels, allowing the facile collection and separation of the products from the electrocatalytic system during the reaction.

**Figure 2.**
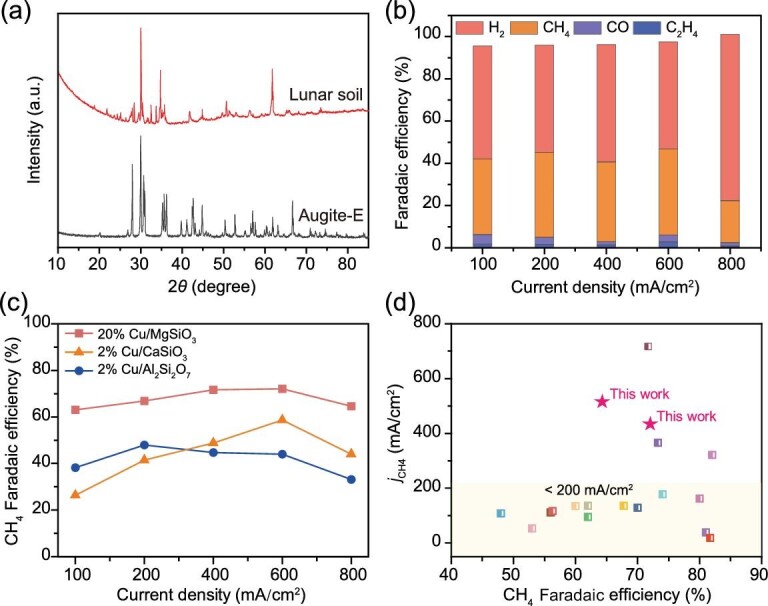
Electrocatalytic CO_2_ conversion performance of the augite-E. (a) Comparison of XRD patterns of lunar soil (CE-5 sample) and augite-E. (b) Faradaic efficiency for H_2_, CH_4_, CO and C_2_H_4_ of Cu/augite-E at different current densities. (c) Faradaic efficiency for H_2_, CH_4_, CO and C_2_H_4_ of optimized Cu/MgSiO_3_ (20% Cu/MgSiO_3_), Cu/CaSiO_3_ (2% Cu/CaSiO_3_) and Cu/Al_2_Si_2_O_7_ (2% Cu/Al_2_Si_2_O_7_) at different current densities. (d) Comparison of the partial current densities and FE of the optimized Cu/MgSiO_3_ reported in this work with those of recently reported catalysts for electrocatalytic CO_2_ conversion.

After demonstrating the high feasibility of our strategy for ISRU of lunar soil toward electrocatalytic CO_2_ conversion for fuel production, we aim for identifying the main active component in lunar soil for optimizing the CH_4_ production. Nevertheless, lunar soil is rather precious and limited on Earth. This limitation motivates us to employ augite on Earth (augite-E; see Supplementary Fig. S3 for details), which has a similar composition and structure to lunar soil, for performing further investigation. To avoid the ambiguities associated with the imitation of lunar soil using augite-E, we first compare their compositions. As shown in Supplementary Figs S4 and S5, the Cu-loaded augite-E (Cu/augite-E) exhibits a bulk structure and its elemental mapping images demonstrate that the chemical composition of our obtained augite-E is similar to that of lunar soil [21]. To further evaluate the imitability of lunar soil by augite-E, we compare their X-ray diffraction (XRD) patterns. As revealed in Fig. 2a, augite-E has a similar crystal structure to lunar soil, allowing evaluation of the feasibility of lunar soil for ISRU using augite-E as an imitant. In addition, after loading of the Cu species on the augite-E, no significant change can be observed in the XRD pattern (Supplementary Fig. S6), suggesting that the Cu species do not alter the phase structures of the augite.

Upon confirming the imitable structure of lunar soil by augite-E, we then employ the Cu/augite-E for electrocatalytic CO_2_ conversion. Although the pristine augite-E demonstrates obvious H_2_ production, no hydrocarbon products can be found in the system, manifesting its poor selectivity toward CO_2_ conversion (Supplementary Fig. S7). In sharp contrast, the Cu/augite-E demonstrates significant production of hydrocarbon fuels (Fig. 2b), further confirming the viability of the ISRU of lunar soil for CO_2_ conversion catalyst preparation. Considering the fact that augite is composed of multiple silicates, we evaluate and compare the electrocatalytic CO_2_ conversion performance of the main silicates in augite (i.e. aluminum silicate (Al_2_Si_2_O_7_, Supplementary Figs S8–S13), calcium silicate (CaSiO_3_, Supplementary Figs S14–S19) and magnesium silicate (MgSiO_3_, Supplementary Figs S20–S28) after loading with Cu species (Supplementary Table S1). Generally, 2% Cu/Al_2_Si_2_O_7_, 2% Cu/CaSiO_3_ and 20% Cu/MgSiO_3_ show the optimal electrocatalytic CO_2_-to-CH_4_ performances (Supplementary Figs S12, S18 and S26). Among them, 20% Cu/MgSiO_3_ exhibits the highest electrocatalytic performance toward CH_4_ production (Fig. 2c), reaching a CH_4_ Faradaic efficiency (FE) of 72.05% at a current density of 600 mA/cm^2^, which enables a CH_4_ production rate of 0.8 mL/min (Supplementary Table S3). It is worth noting that the FE_CH4_ is 64.59% at 800 mA/cm^2^, corresponding to a maximum CH_4_ partial current density of 516.7 mA/cm^2^. Such a result is obtained because 20% Cu/MgSiO_3_ exhibits superior reaction kinetics (Supplementary Fig. S29) and electron-transfer rates (Supplementary Figs S30 and S31) for electrochemical CO_2_ conversion. As such, it can be confirmed that the MgSiO_3_ is the main active component of augite in initiating the CH_4_ production after loading with Cu species. Such a result suggests that the precise tuning of the composition of the lunar soil (i.e. increasing the ratio of the MgSiO_3_) can result in enhanced production of hydrocarbon fuels during the electrocatalytic CO_2_ conversion. What is more, the CH_4_ FE obtained through electrocatalytic CO_2_ conversion using our prepared samples is comparable with the performance of commonly applied electrocatalysts such as Cu-based metal–organic frameworks [22,23], Cu/CeO*_x_*[24] and Cu/Al_2_O_3_ [25] (see Fig. 2d)and Supplementary Table S4), conclusively demonstrating that the ISRU of lunar soil for catalyst synthesis is highly feasible on the Moon. In addition, we also investigate oxygen evolution, which is another critical survival necessity for humans, in the system. Remarkably, superior oxygen production can be also obtained from the system, reaching a production rate of 2.3 mL/min with a current density of 600 mA/cm^2^ (Supplementary Fig. S32). Based on these results, it can be confirmed that MgSiO_3_ is the main active CH_4_ production component in lunar soil, which can be loaded with Cu species to achieve outstanding electrocatalytic CO_2_ conversion performance.

### Electrocatalytic CO_2_ conversion mechanism

**Figure 3.**
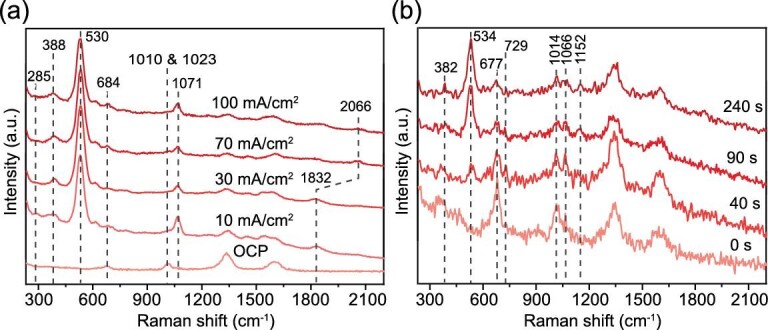
Electrocatalytic CO_2_ conversion mechanism. (a) *In situ* Raman spectra of the electrocatalytic CO_2_ conversion test using optimized Cu/MgSiO_3_ under different current densities. (b) *In situ* Raman spectra of the electrocatalytic CO_2_ conversion test using optimized Cu/MgSiO_3_ with the evolution of the reaction duration.

To have a full image of the electrocatalytic mechanism and performance of lunar soil, we perform *in situ* Raman spectroscopy characterizations (see Supplementary Fig. S33 for experimental set-up) for discerning the intermediates formed during the electrocatalytic CO_2_ conversion using Cu/MgSiO_3_. As shown in Fig. 3a, under the open-circuit potential, two main peaks at 684 and 1023 cm^−1^ assigned to the silicates can be observed [26]. With the increase in the current density, these two peaks gradually decrease and a peak at 1071 cm^−1^ attributed to the adsorbed carbonate appears. In addition, a strong peak at 530 cm^−1^ attributed to the Cu–O–Si can be also found after supplying the current density to the system [27], suggesting the strong interaction between Cu species and MgSiO_3_ during the reaction. Furthermore, a peak at 1832 cm^−1^ attributed to the *CO on the Cu appears, implying the reduction of Cu species into Cu nanoparticles during the reaction [28]. This peak gradually decreases with the increasing current density and a peak at 2066 cm^−1^ attributed to the adsorbed CO on top of terrace-like sites of Cu emerges, which is beneficial for the reduction reaction for CH_4_ production [27]. Two minor peaks at 285 and 388 cm^−1^ assigned to the Cu–CO can be also observed [29], further confirming the roles of Cu in activating CO_2_ and stabilizing *CO for the subsequent reduction reaction. A similar trend can be also observed by performing *in situ* Raman characterization for the electrocatalytic CO_2_ conversion with the evolution of time (see Fig. 3b), corroborating the critical functions of Cu in such a reaction.

### Unmanned and scalable fuel and oxygen production

**Figure 4.**
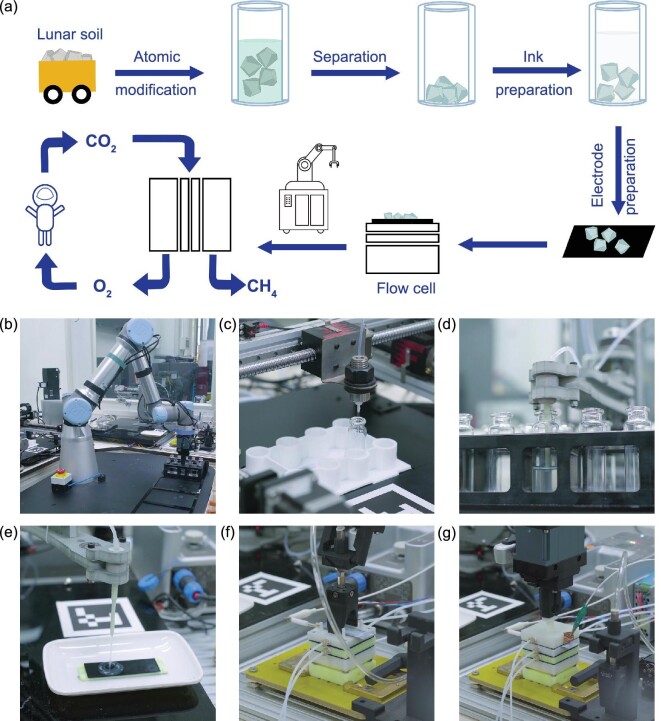
Unmanned lunar soil utilization for electrocatalytic CO_2_ conversion. (a) Schematic illustration of the set-up and procedures for unmanned lunar soil utilization for electrocatalytic CO_2_ conversion. (b–g) Photograph showing the robotic system gripping the vial filled with silicate from the tube rack to the dosing station (b), filling the vial with the cupric chloride solution at the dosing station (c), removing the upper suspension of the mixed solution at the pipetting station (d), dropping catalyst ink onto the carbon paper (e), placing the dried carbon paper with catalyst onto the flow cell (f), and assembling the flow cell for electrocatalytic CO_2_ conversion test (g).

Given that the ultimate aim of the strategy reported in this work is to build up a large-scale unmanned electrocatalytic fuel and oxygen production system, the participation of the robotic system in the electrocatalytic CO_2_ conversion is highly desirable. To this end, we have developed a robotic system for electrocatalytic CO_2_ conversion (Fig. 4a), which is enabled by the full accessibility of our developed process. Such a robotic system can collect and process the lunar soil (Fig. 4b), achieving the unmanned ISRU of lunar soil for catalyst production. As a following step, it can also perform the materials preparation for loading Cu on the lunar soil (Fig. 4c). Finally, the electrocatalytic system can be automatically set up by our developed robotic system, including catalyst ink preparation (Fig. 4d), electrode preparation (Fig. 4e) and flow-cell set-up (Fig. 4f). No significant differences can be found between the manned and unmanned electrocatalytic tests (Fig. 4g), implying the high feasibility of the robotic system for operating the electrocatalytic CO_2_ conversion. Such an achievement again underscores that our proposed unmanned electrocatalytic CO_2_ conversion strategy (Supplementary Movie 1) can be easily imitated on extraterrestrial sites. Another noteworthy perspective for such an unmanned system is the feasibility of *in situ* screening catalyst recipes based on lunar soil to achieve optimal performance in the future, given the varied compositions of lunar soil in different regions.

## CONCLUSION

In short, we have comprehensively demonstrated the practicability of *in situ* lunar soil utilization for highly efficient extraterrestrial fuel and oxygen supply via electrocatalytic CO_2_ conversion. After loading with Cu species, the selected silicate from lunar soil (i.e. MgSiO_3_) can reach a methane production rate of 0.8 mL/min at a current density of 600 mA/cm^2^. Concurrently, oxygen can be produced with a production rate of 2.3 mL/min. With such a superior performance, this system is expected to supply sufficient fuel and oxygen during the extraterrestrial mission after scaling up. More importantly, we have demonstrated the full accessibility of our catalyst preparation process and developed a robotic system for achieving unmanned electrocatalytic CO_2_ conversion. No significant difference can be observed between the manned and unmanned systems, which further suggests the high possibility of imitating our proposed system in extraterrestrial sites and proves the feasibility of further optimizing catalyst recipes on the Moon. The study in this work can not only be regarded as a major step forward in ISRU of lunar soil, but also shines light on the fuel and oxygen production system for the sustainable supply of critical survival necessities on extraterrestrial sites. The findings on catalyst design in this work also provide important insights for the development of highly efficient electrocatalytic materials toward CO_2_ conversion on Earth.

## METHODS

### Preparation of Cu/lunar soil

Typically, 100 mL of CuCl_2_·2H_2_O solution (10 mg/mL) was first prepared by dissolving 1 g of CuCl_2_·2H_2_O in deionized (DI) water (18.2 MΩ cm). The solution was transferred to a 100-mL volumetric flask and diluted to the calibration mark with DI water. Subsequently, the weight ratio of CuCl_2_·2H_2_O to the lunar soil was set to 500% by ultrasonically dispersing 10 mg of lunar soil in 5 mL of CuCl_2_·2H_2_O solution (10 mg/mL) for 1 h. Finally, Cu/lunar soil was collected by centrifugation and washed with ultrapure water. Cu/augite-E, Cu/Al_2_Si_2_O_7_, Cu/CaSiO_3_ and Cu/MgSiO_3_ with different weight ratios of CuCl_2_·2H_2_O (see also Supplementary Table S1) were prepared by a similar procedure by swapping the lunar soil for augite-E, Al_2_Si_2_O_7_, CaSiO_3_ and MgSiO_3_, respectively.

### Electrochemical measurements

All electrochemical measurements were performed in a three-channel flow cell in aqueous 1 M KOH as shown in Supplementary Fig. S1. The electrochemical measurements were controlled by an electrochemical workstation (CHI 660e) equipped with a current amplifier (CHI 680c). To prepare the working electrode, 2.5 mg of catalyst was dispersed by sonication in the mixture of isopropanol (970 μL) and Nafion ionomer solution (5%, Sigma-Aldrich) (30 μL) for 30 min. Subsequently, 180 μL of the catalyst ink was dropped onto a gas diffusion layer (YLS 30T, Fuel Cell Store) as the cathode electrode (1 × 1 cm^2^). A saturated Ag/AgCl electrode was used as the reference electrode. An anion exchange membrane (FAB-PK-130, Fuel Cell Store) was sandwiched between the anode (nickel foam) and cathode. 1 M KOH was circulated in the anolyte and catholyte chambers at a flow rate of 10 mL/min during CO_2_ electrolysis. The high-purity CO_2_ or argon (Ar) (Linde, 99.999%) gas flowed through the porous gas diffusion layer (GDL) to the catalyst layer in contact with the bulk electrolyte, forming the gas–electrolyte–catalyst triple-phase interface. The gas flow rate was set to 50 sccm via a mass flow controller (D08-1F, Sevenstar). An LSV experiment of 500% Cu/lunar soil was performed in CO_2_ and Ar environments at a scan rate of 50 mV/s with 85% iR compensation.

The cathodic gaseous products were analysed using gas chromatography (GC, 7890A and 7890B, Agilent) and the cathodic liquid products were analysed by ^1^H nuclear magnetic resonance spectroscopy (Bruker AVANCE AVIII 400). The production rate of anodic product (i.e. O_2_) was detected using an electronic soap film flowmeter (JCL-2010, Qingdao Juchuang Environmental Protection Group). All potentials were converted to the reversible hydrogen electrode in scale according to the following equation:

\begin{eqnarray*} E\left( {{\rm{vs}}{\rm{. RHE}}} \right) &=& E\left( {{\rm{vs}}{\rm{. Ag/AgCl}}} \right)\\ &&+\, 0{\rm{.197 + 0}}{\rm{.0591 \times pH}}. \end{eqnarray*}

The FE of the CO_2_ electrolysis products was calculated using the following equation:

\begin{eqnarray*} {\rm{FE }}\left( {\rm{\% }} \right) &=& \frac{Q}{{{Q}_{total}}} \times 100{\rm{\% }}\\ &=& \frac{{{n}_e \times n \times F}}{{{Q}_{total}}} \times 100{\rm{\% }}, \end{eqnarray*}

where *Q* and *Q_total_* represent the charges transferring into the corresponding product and the total charge passed through the cathode during the electrolysis, respectively; *F* represents the Faraday constant (96485 C/mol); *n* is the mole amount of the corresponding product; and *n_e_* is the number of electrons transferred.

### *In situ* Raman spectroscopy

*In situ* Raman spectra were recorded on a WITec Alpha 300R Raman spectrometer with a 633-nm laser as the excitation light source. The experiments were performed in a self-made flow cell as shown in Supplementary Fig. S33. CO_2_ gas was introduced to the back of the GDL at a flow rate of 50 sccm controlled by a rotameter. The CO_2_ electrolysis was controlled by chronopotentiometry. The current was increased gradually while *in situ* Raman spectra were recorded.

## Supplementary Material

nwac200_Supplemental_Files
