# Supplementary material for: In situ resource utilization of lunar soil for highly efficient extraterrestrial fuel and oxygen supply
Source: Natl Sci Rev. 2022 Sep 23;10(2):nwac200. doi: 10.1093/nsr/nwac200 (PMC9935986; doi:10.1093/nsr/nwac200)
Supplement: nwac200_Supplemental_Files [file nwac200_supplemental_files.zip › Supplementary data.pdf]

Supplementary information for

In situ resource utilization of lunar soil for highly efficient extraterrestrial fuel and oxygen supply

Yuan Zhong[1,†], Jingxiang Low[1,†], Qing Zhu[1,†], Yawen Jiang[1], Xiwen Yu[2], Xinyu Wang[1], Fei Zhang[1], Weiwei Shang[1], Ran Long[1,*], Yingfang Yao[2,*], Wei Yao[3], Jun Jiang[1,*], Yi Luo[1], Weihua Wang[3], Jinlong Yang[1], Zhigang Zou[2,*] and Yujie Xiong[1,*]

[1]Hefei National Research Center for Physical Sciences at the Microscale, School of Chemistry and Materials Science, National Synchrotron Radiation Laboratory, School of Information Science and Technology, University of Science and Technology of China, Hefei, Anhui 230026, China;
[2]Eco-Materials and Renewable Energy Research Center (ERERC), Jiangsu Key Laboratory for Nano Technology, National Laboratory of Solid State Microstructures, School of Physics, Collaborative Innovation Center of Advanced Microstructures, College of Engineering and Applied Sciences, Nanjing University, Nanjing 210093, China;
[3]Qian Xuesen Laboratory of Space Technology, China Academy of Space Technology, Beijing 100094, China

***Corresponding authors.** E-mails: longran@ustc.edu.cn; yaoyingfang@nju.edu.cn; jiangj1@ustc.edu.cn; zgzou@nju.edu.cn; yjxiong@ustc.edu.cn

[†]Equally contributed to this work.

Experimental Procedures

Chemicals. Copper (II) chloride ($CuCl_2 \cdot 2H_2O$, 99%), potassium hydroxide (KOH, ≥85%), ethanol and isopropanol were purchased from Sinopharm Chemical Reagent Co. Ltd. (Shanghai, China). Augite-E was mined from Dandong, China. Augite-E was ground into powder through a grinding machine (JXFSTPRP-32L, Shanghai Jingxin), and sieved by a 200-mesh sieve for further use. Florisil ($2MgO \cdot 3SiO_2 \cdot nH_2O$, denoted as $MgSiO_3$) and calcium silicate ($CaSiO_3$) were purchased from Aladdin Chemical Co., Ltd., and aluminum silicate ($Al_2Si_2O_7 \cdot 2H_2O$) was purchased from Alfa Aesar Chemical Co., Ltd. All the reagents were commercially available and used as received without further purification.

Materials Characterization

Transmission electron microscopy (TEM) images were acquired on a Hitachi 7700 transmission electron microscope. Energy-dispersive X-ray spectroscopy (EDS) mapping profiles were collected using JEOL JEM-F200 and JEM-2100F transmission electron microscope. Scanning electron microscopy (SEM) was performed on a Gemini SEM 500. Powder X-ray diffraction (XRD) was performed on a Philips X'Pert Pro Super X-ray diffractometer (Netherlands) with Cu Kα radiation (λ=1.5406 Å). X-ray absorption fine structure spectra (XAFS) measurements were performed at the beamline 1W1B in Beijing Synchrotron Radiation Facility (BSRF), China. X-ray photoelectron spectra (XPS) were collected on an ESCA Lab 250 X-ray photoelectron spectrometer (USA), using monochromatic Al Kα X-ray (1486.6 eV) as the excitation

source. The bulk electrical resistivity was acquired on the resistivity tester (ST2722, Suzhou Jingge).

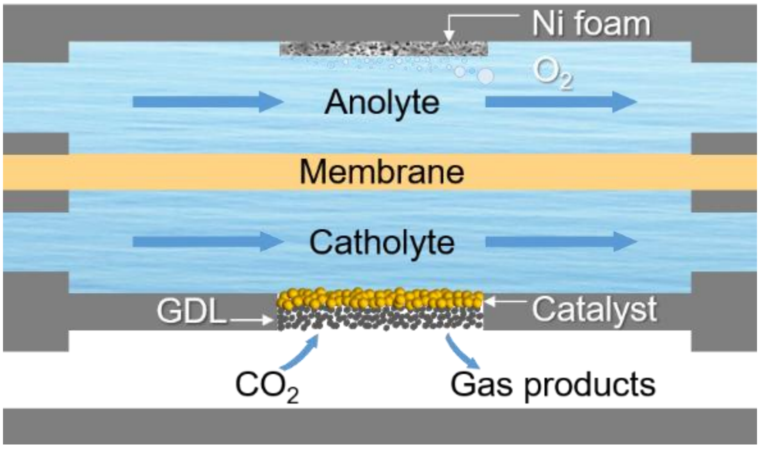

Figure S1. Schematic diagram of the flow cell for electrocatalytic CO_2 conversion.

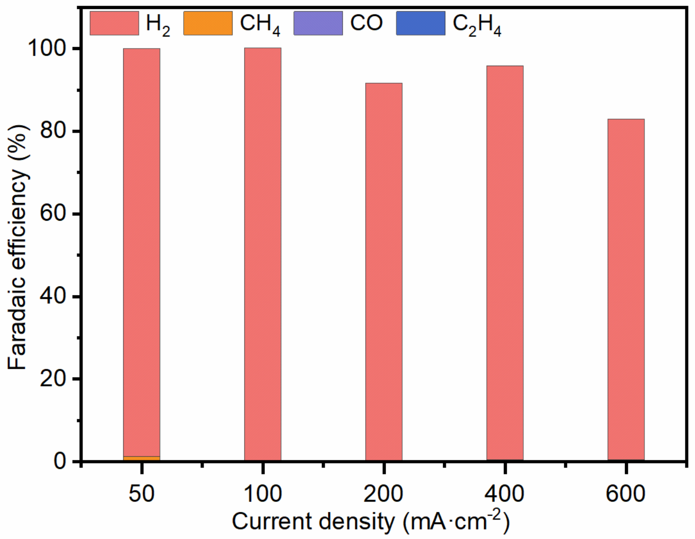

Figure S2. Electrochemical CO_2 reduction reaction (CO_2RR) performance of Chang'E-5 lunar soil. By using the pristine CE-5 sample as a catalyst, H_2 is the major product while negligible hydrocarbon products can be detected.

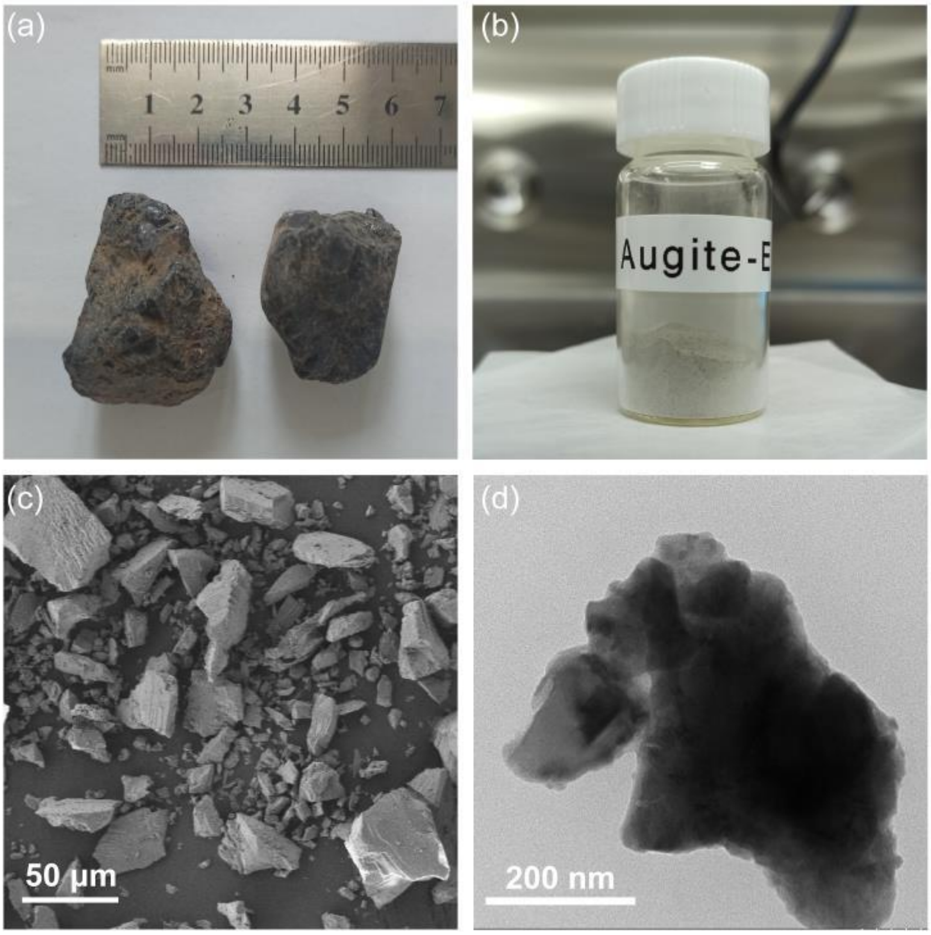

Figure S3. (a and b) Photographs of the Augite-E before (a) and after (b) milling. (c and d) SEM (c) and TEM (d) images of the Augite-E after milling.

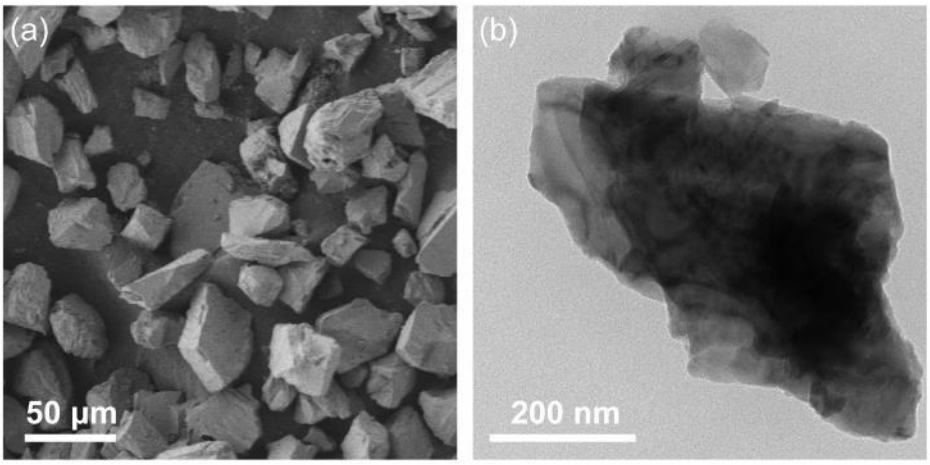

Figure S4. (a) SEM and (b) TEM images of 500% Cu/Augite-E.

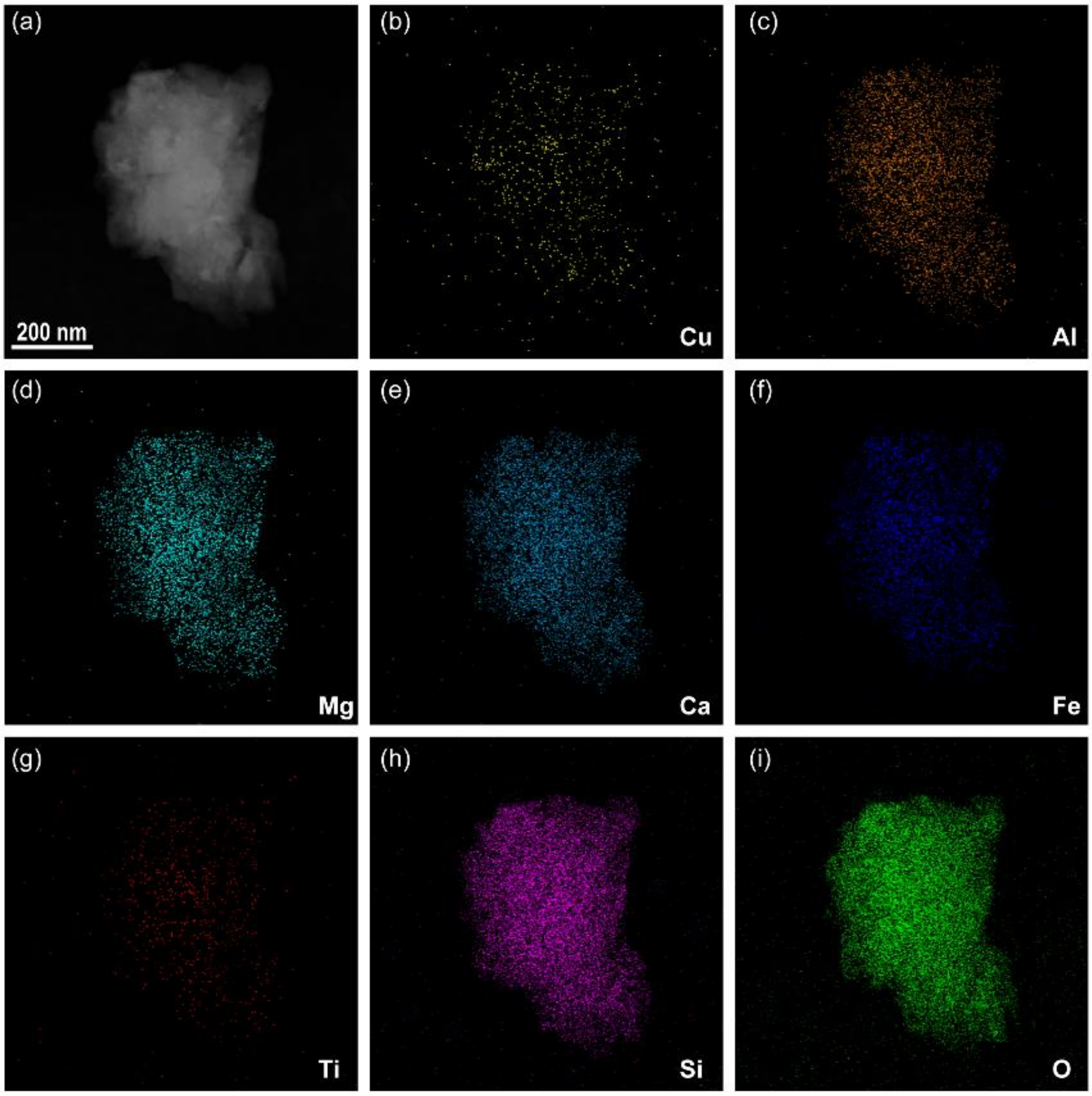

Figure S5. Elemental mapping images of the 500% Cu/Augite-E catalyst for Cu, Al, Ca, Mg, Fe, Ti, Si and O elements. The elemental mapping of the 500% Cu/Augite-E shows the uniform distribution of Cu elements on the Augite-E support.

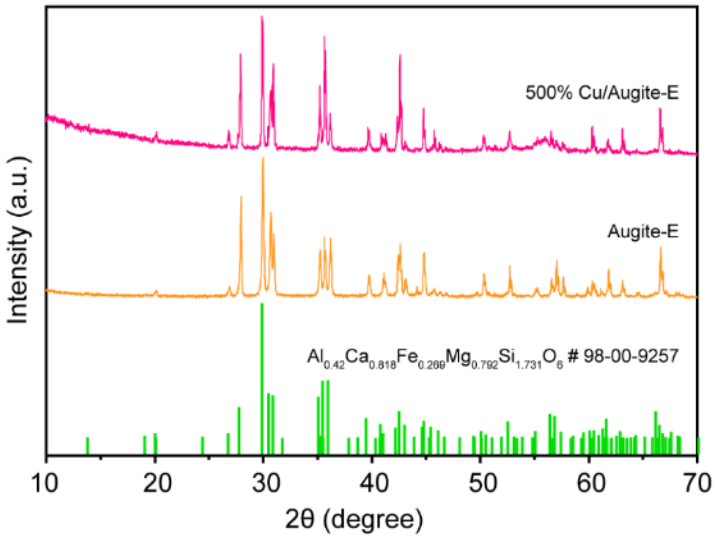

Figure S6. Comparison of XRD patterns of the Augite-E and 500% Cu/Augite-E. No obvious change can be observed between the Augite-E and 500% Cu/Augite-E, suggesting that the introduction of Cu species does not alter the phase structure of the Augite-E.

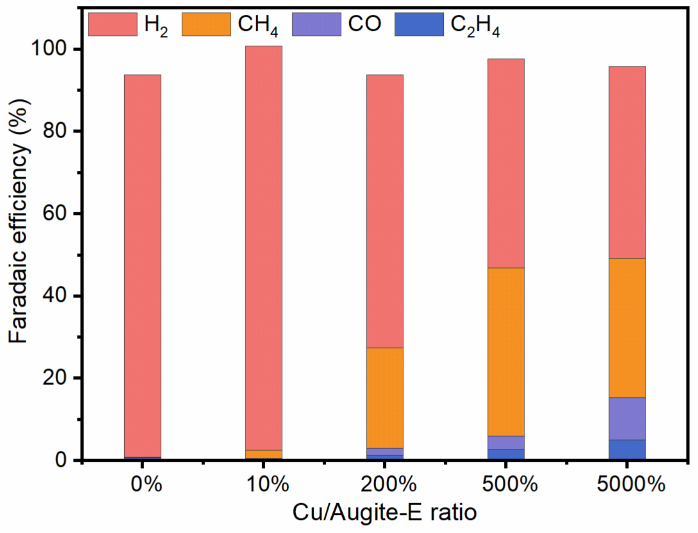

Figure S7. Electrochemical CO_2RR performance comparison of Cu/Augite-E catalysts with different Cu/Augite-E ratios at a current density of 600 mA/cm^2.

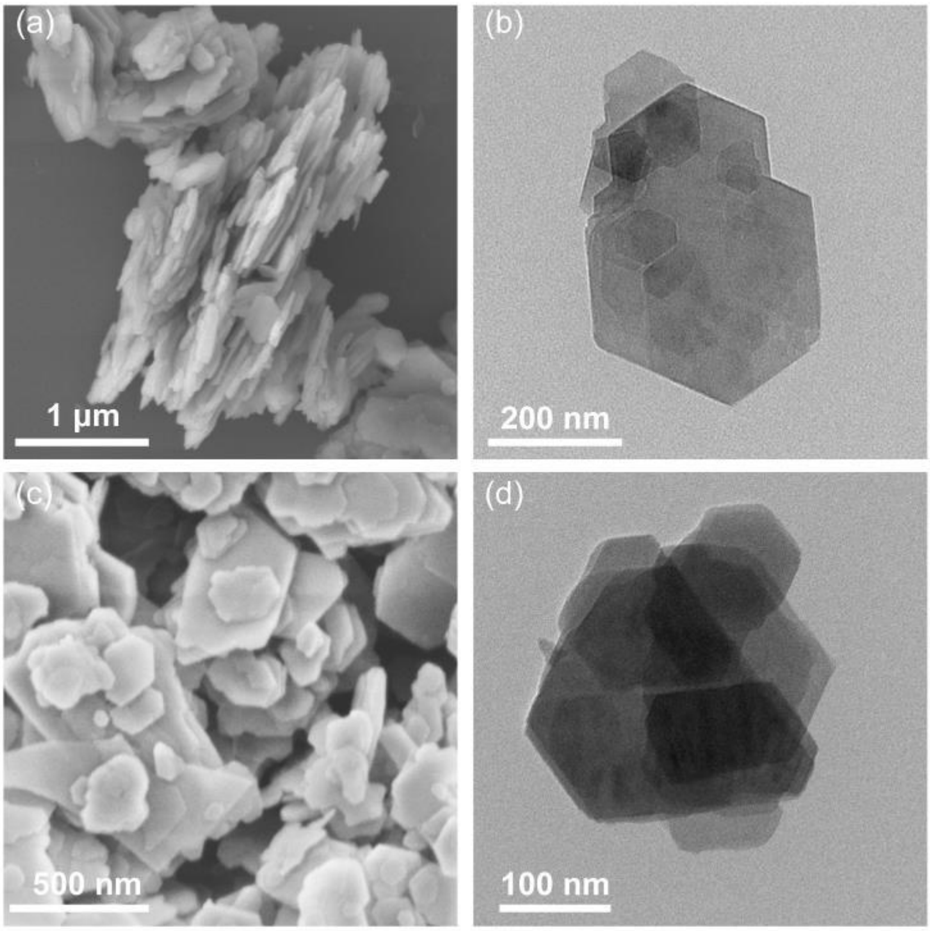

Figure S8. SEM and TEM images of $Al_2Si_2O_7$ (a and b) and 2% Cu/$Al_2Si_2O_7$ (c and d). The SEM and TEM images show that 2% Cu/$Al_2Si_2O_7$ inherits the hexagonal nanosheet-like morphology of $Al_2Si_2O_7$.

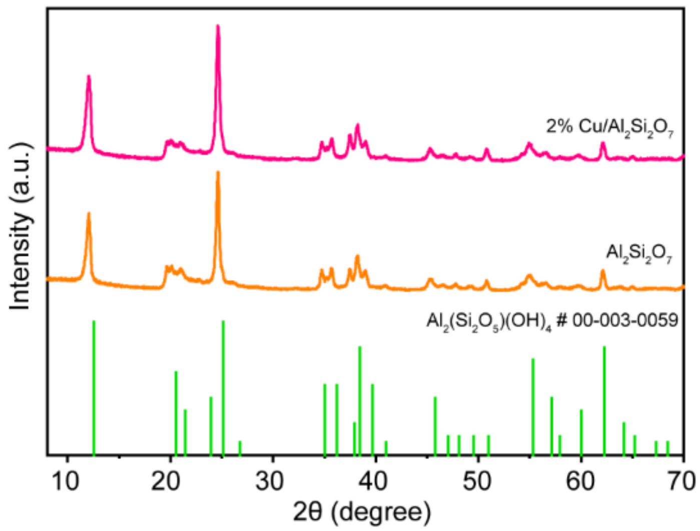

Figure S9. Comparison of XRD patterns of the $Al_2Si_2O_7$ and 2% Cu/$Al_2Si_2O_7$. No obvious change can be observed between the $Al_2Si_2O_7$ and 2% Cu/$Al_2Si_2O_7$, suggesting that the introduction of Cu species does not alter the phase structure of the $Al_2Si_2O_7$.

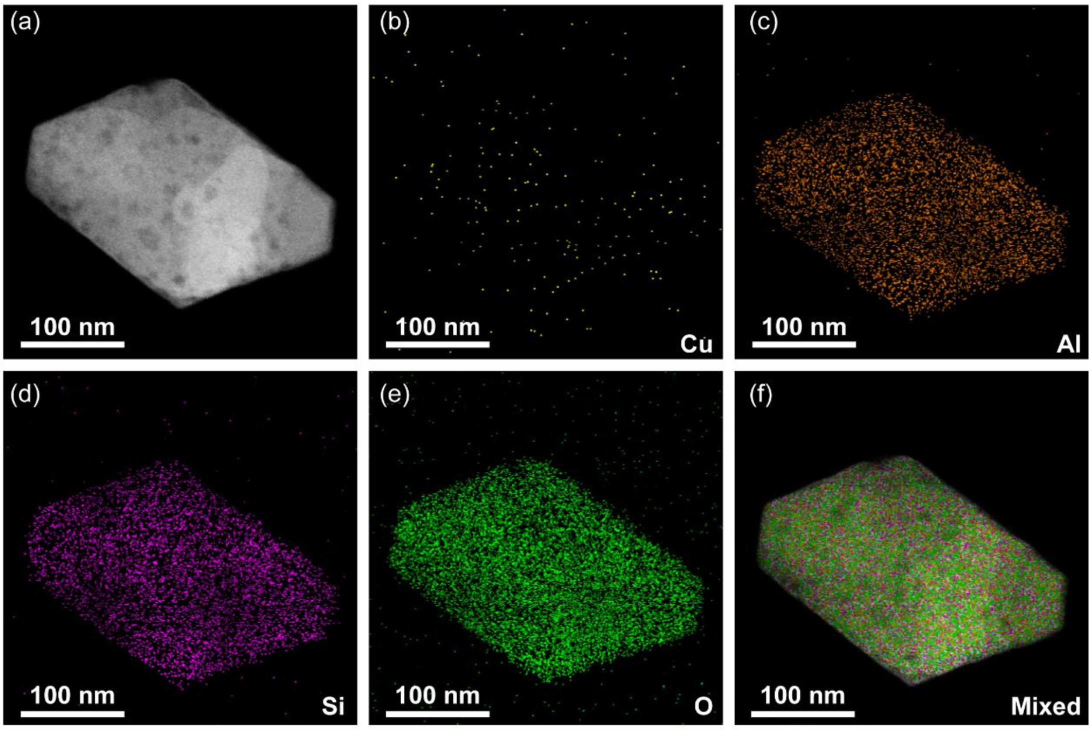

Figure S10. Elemental mapping images of 2% Cu/$Al_2Si_2O_7$ for Cu, Al, Si and O elements. The elemental mapping of 2% Cu/$Al_2Si_2O_7$ shows the uniform distribution of Cu elements on the $Al_2Si_2O_7$ support.

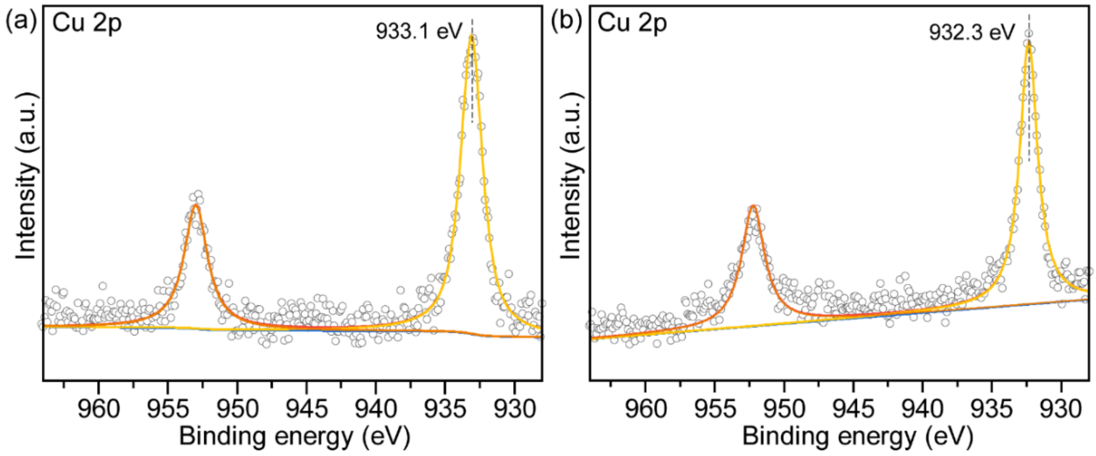

Figure S11. (a) High-resolution Cu 2p XPS spectra of 2% Cu/$Al_2Si_2O_7$. The Cu $2p_{3/2}$ peak at 933.1 eV suggests the ionic $Cu^{\delta+}$ ($0 < \delta < 2$) characteristics of copper adsorbed on 2% Cu/$Al_2Si_2O_7$. (b) High-resolution Cu 2p XPS spectra of 2% Cu/$Al_2Si_2O_7$ after electrolysis. During the CO_2RR electrolysis, the Cu species adsorbed on $Al_2Si_2O_7$ is reduced to metallic Cu, resulting in the shift of the Cu 2p peak.

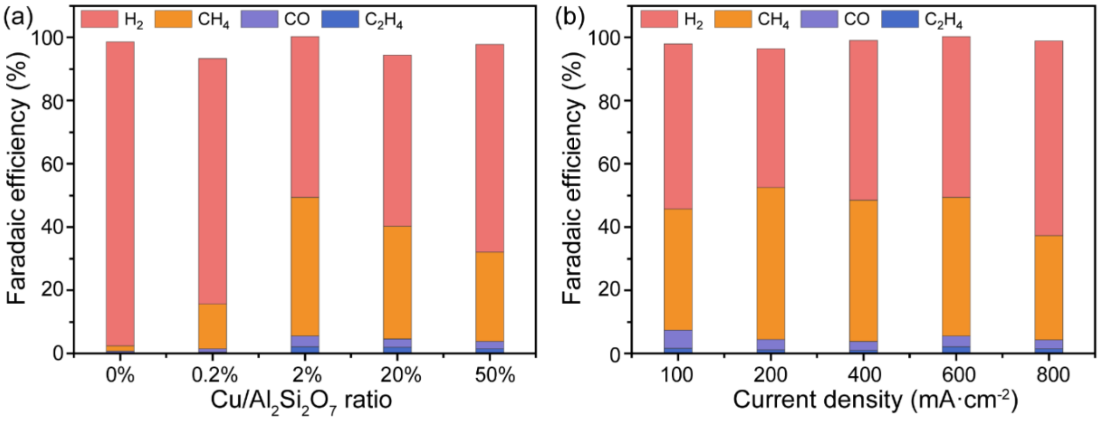

Figure S12. (a) The major CO_2RR product distribution of catalysts with different Cu/$Al_2Si_2O_7$ ratios at a current density of 600 mA/cm^2. (b) The Faradaic efficiency for CO_2RR products of 2% Cu/$Al_2Si_2O_7$ catalysts under different current densities.

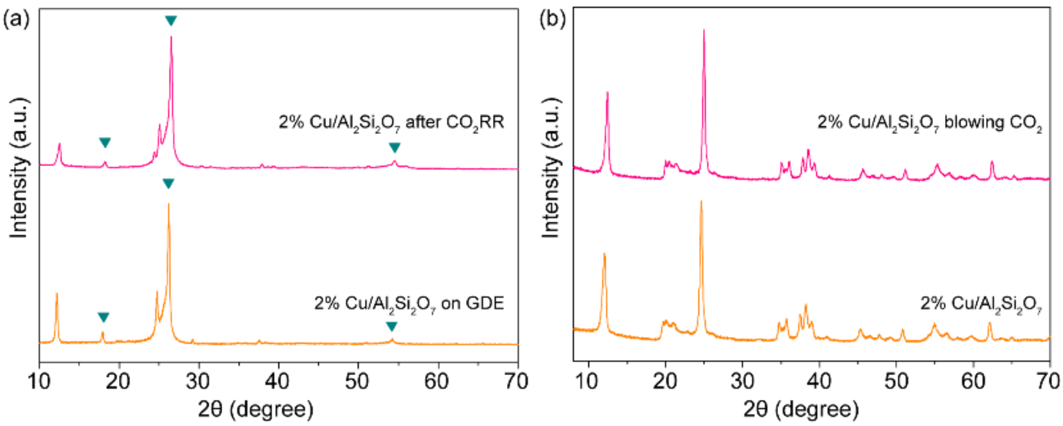

Figure S13. (a) Comparison of XRD patterns of 2% $Cu/Al_2Si_2O_7$ before and after CO_2RR. The XRD profiles of the blank gas diffusion electrode (GDE) are marked with inverted triangles. (b) Comparison of XRD patterns of 2% $Cu/Al_2Si_2O_7$ before and after blowing CO_2. No significant changes can be observed in the 2% $Cu/Al_2Si_2O_7$ after undergoing various reaction environments, suggesting its high stability.

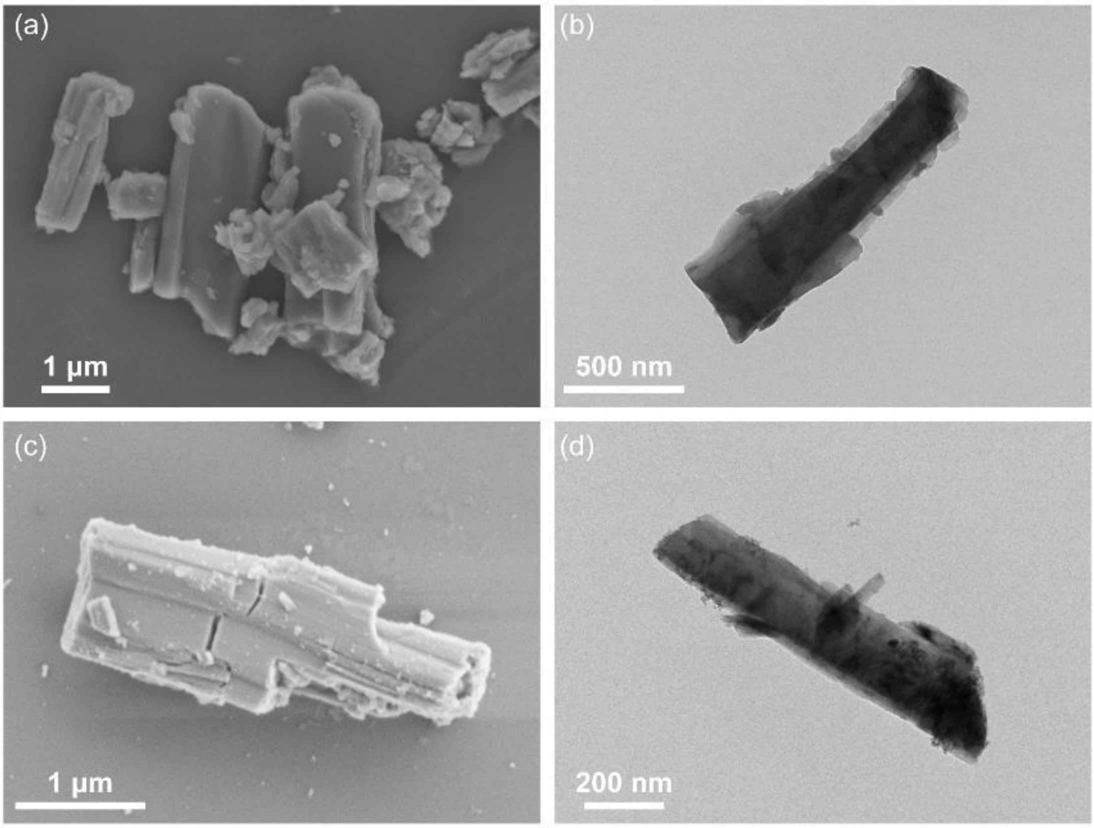

Figure S14. SEM and TEM images of $CaSiO_3$ (a and b) and 2% $Cu/CaSiO_3$ (c and d). The SEM and TEM images show that 2% $Cu/CaSiO_3$ inherits the rod-like morphology of $CaSiO_3$.

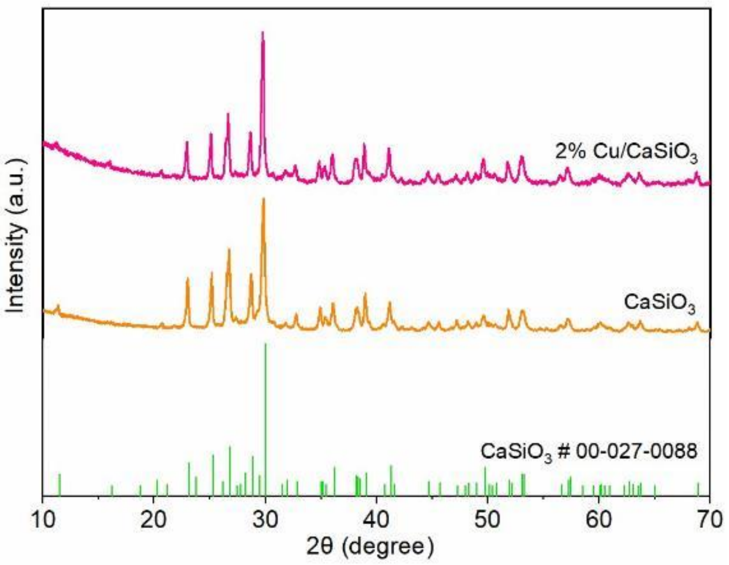

Figure S15. Comparison of XRD patterns of the $CaSiO_3$ and 2% Cu/$CaSiO_3$. No obvious change can be observed between the $CaSiO_3$ and 2% Cu/$CaSiO_3$, suggesting that the introduction of Cu species does not alter the phase structure of the $CaSiO_3$.

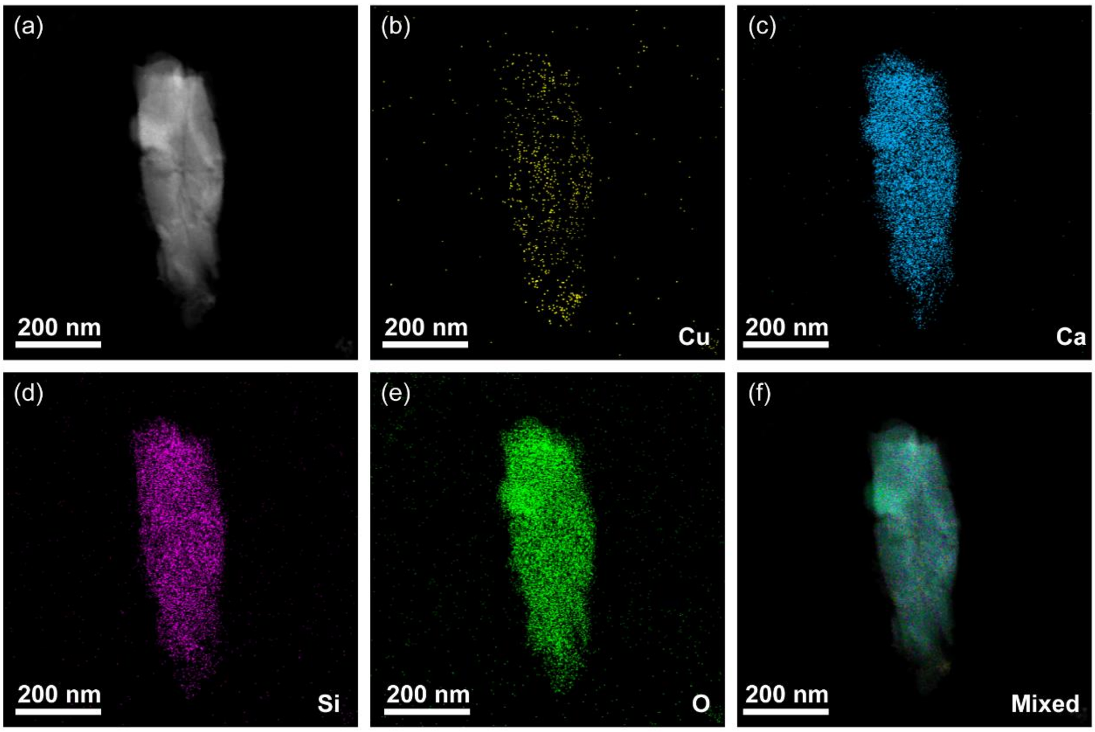

Figure S16. Elemental mapping images of 2% Cu/$CaSiO_3$ for Cu, Ca, Si and O elements. The elemental mapping of 2% Cu/$CaSiO_3$ shows the uniform distribution of Cu elements on the $CaSiO_3$ support.

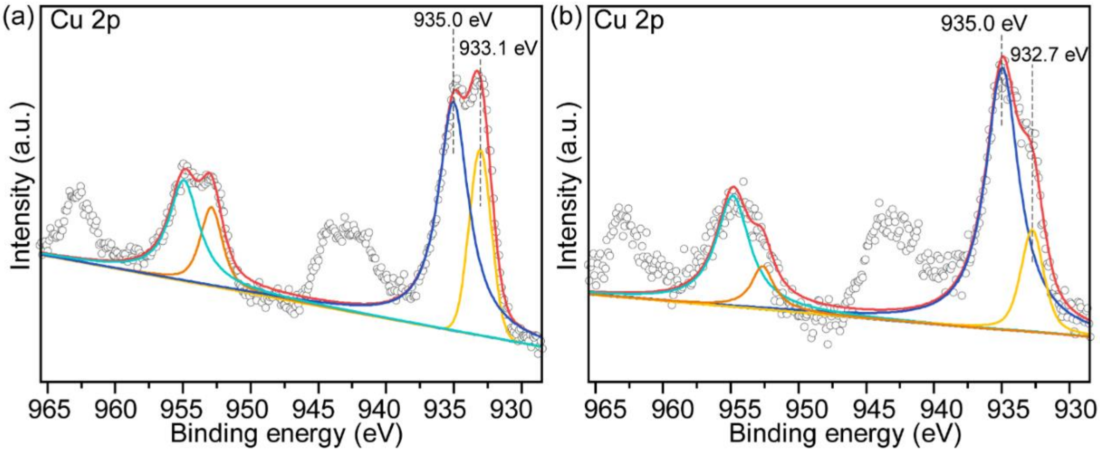

Figure S17. (a) High-resolution Cu 2p XPS spectra of 2% Cu/$CaSiO_3$. The Cu $2p_{3/2}$ peak at 933.1 eV suggests the ionic $Cu^{\delta+}$ ($0 < \delta < 2$) characteristics of copper adsorbed on 2% Cu/$CaSiO_3$. The Cu $2p_{3/2}$ peak at 935.0 eV can be assigned to $CuSiO_3$. (b) High-resolution Cu 2p XPS spectra of 2% Cu/$CaSiO_3$ after electrolysis.

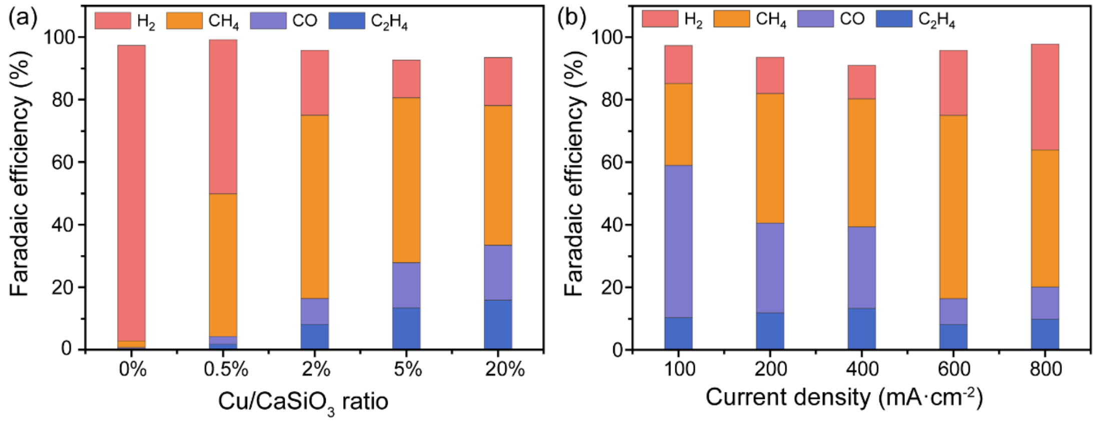

Figure S18. (a) The major CO_2RR product distribution of catalysts with different Cu/$CaSiO_3$ ratios at a current density of 600 mA/cm^2. (b) The Faradaic efficiency for CO_2RR products of 2% Cu/$CaSiO_3$ catalysts under different current densities.

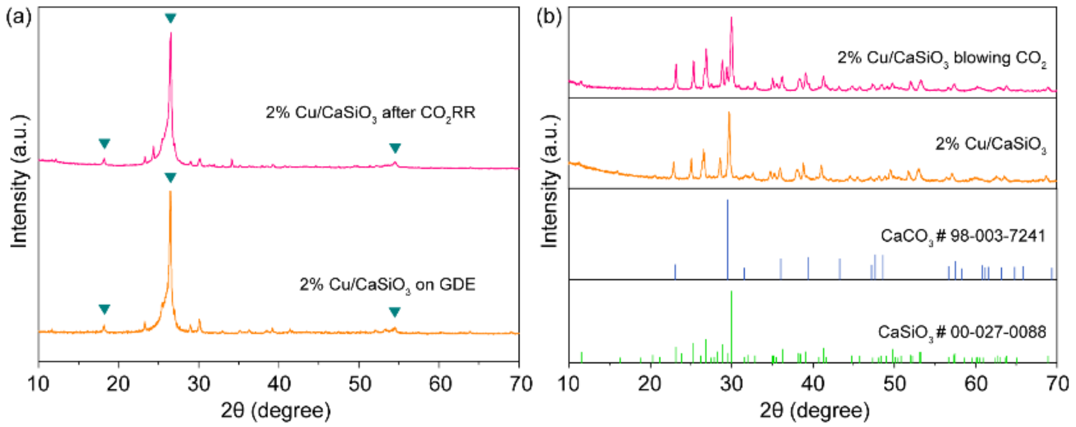

Figure S19. (a) Comparison of XRD patterns of 2% Cu/$CaSiO_3$ before and after CO_2RR. The XRD profiles of blank GDE are marked with inverted triangles. (b) Comparison of XRD patterns of 2% Cu/$CaSiO_3$ before and after blowing CO_2. After undergoing the CO_2RR reaction, a new peak at 29.5 ° attributed to $CaCO_3$ can be found on the sample, implying that part of the $CaSiO_3$ is transformed into the $CaCO_3$ during the electrochemical CO_2RR.

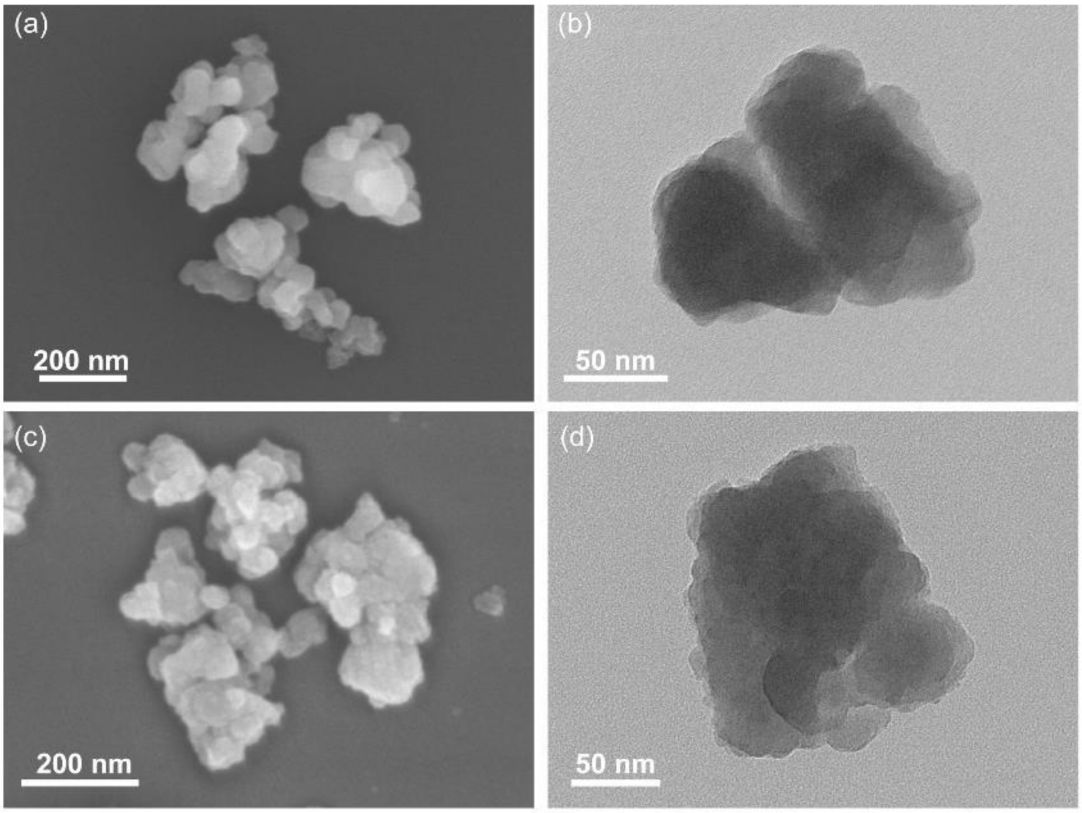

Figure S20. SEM and TEM images of $MgSiO_3$ (a and b) and 20% Cu/$MgSiO_3$ (c and d). The SEM and TEM images show that 20% Cu/$MgSiO_3$ inherits the morphology of $MgSiO_3$.

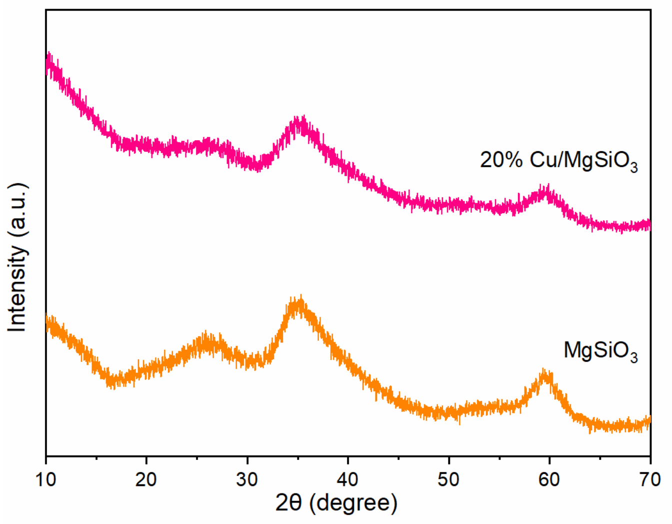

Figure S21. Comparison of XRD patterns of the $MgSiO_3$ and 20% Cu/$MgSiO_3$. No obvious change can be observed between the $MgSiO_3$ and 20% Cu/$MgSiO_3$, suggesting that the introduction of Cu species does not alter the phase structure of the $MgSiO_3$.

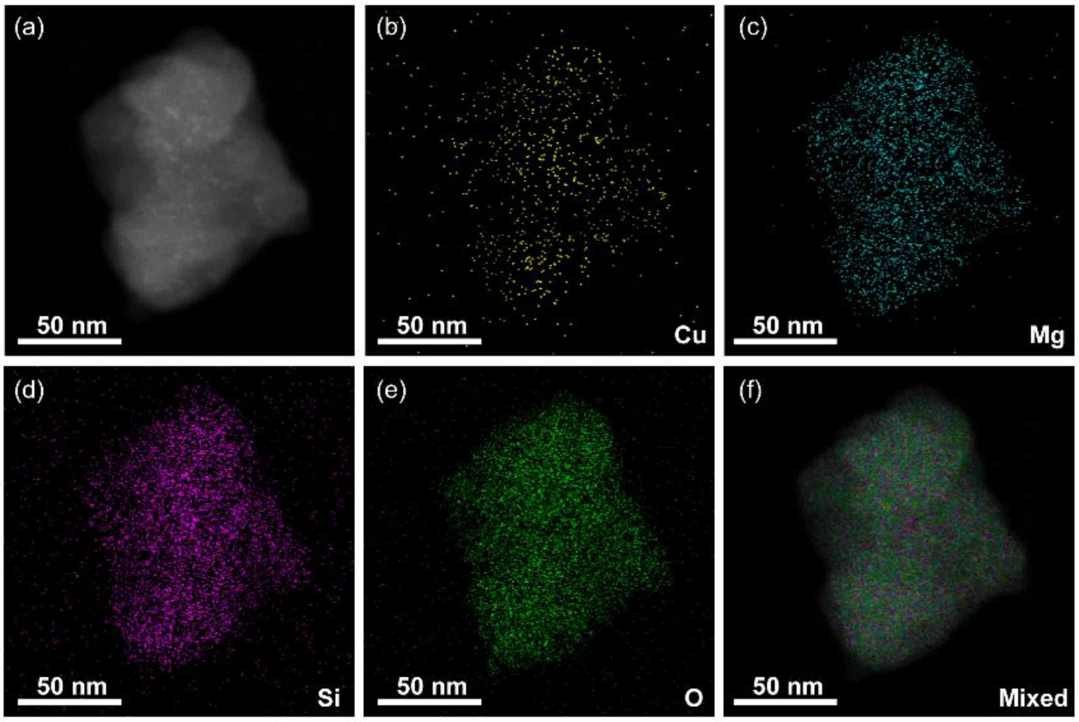

Figure S22. Elemental mapping images of 20% Cu/$MgSiO_3$ for Cu, Mg, Si and O elements. The elemental mapping of 20% Cu/$MgSiO_3$ shows the uniform distribution of Cu elements on the $MgSiO_3$ support.

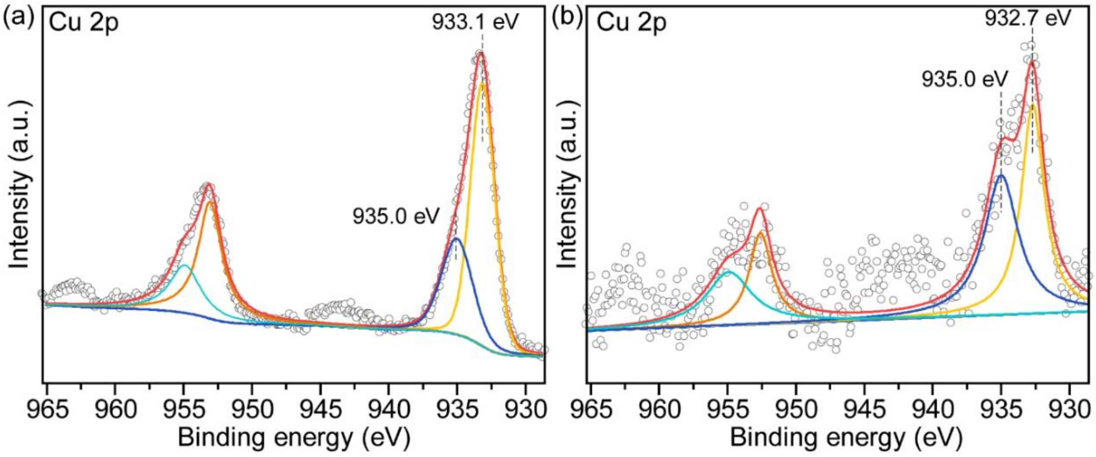

Figure S23. (a) High-resolution Cu 2p XPS spectra of 20% Cu/$MgSiO_3$. The Cu $2p_{3/2}$ peak at 933.1 eV suggests the ionic $Cu^{\delta+}$ ($0 < \delta < 2$) characteristics of copper adsorbed on 20% Cu/$MgSiO_3$. The Cu $2p_{3/2}$ peak at 935.0 eV can be assigned to $CuSiO_3$. (b) High-resolution Cu XPS spectra of 20% Cu/$MgSiO_3$ after electrolysis. For the 20% Cu/$MgSiO_3$, the peak assigned to $CuSiO_3$ significantly increases after electrocatalytic CO_2 conversion, suggesting the increase in $CuSiO_3$ content. Such an increase in $CuSiO_3$ content is beneficial for enhancing electrocatalytic CO_2 conversion because Cu^{2+} species can promote the adsorption of *CO for enhancing the selectivity of the reaction toward CH_4 production [3].

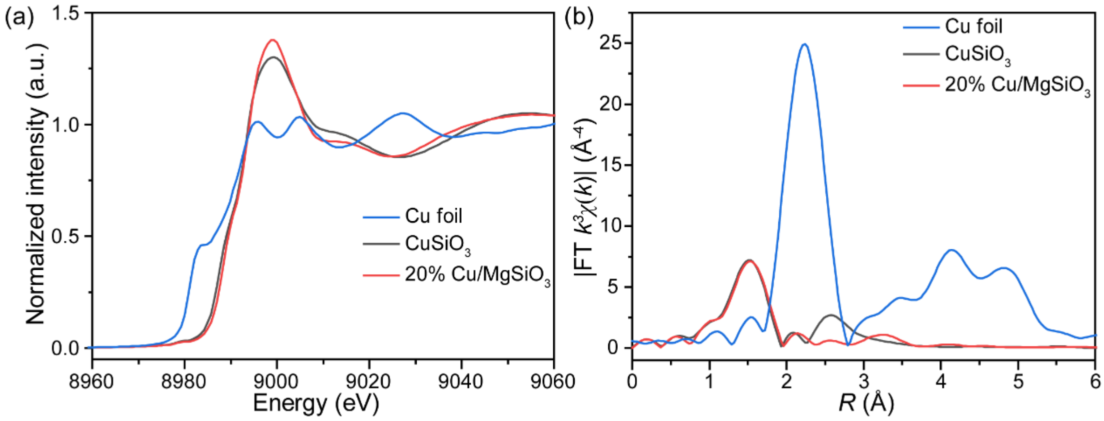

Figure S24. (a) XANES spectra at the Cu K-edge of 20% Cu/$MgSiO_3$, $CuSiO_3$ and Cu foil, and (b) corresponding k^3-weighted Cu K-edge EXAFS spectra. As shown in X-ray absorption near edge structure (XANES) spectra (Fig. S24a), the pre-edge peaks of 20% Cu/$MgSiO_3$ are similar to that of $CuSiO_3$, suggesting the presence of $CuSiO_3$ on the 20% Cu/$MgSiO_3$ [1].

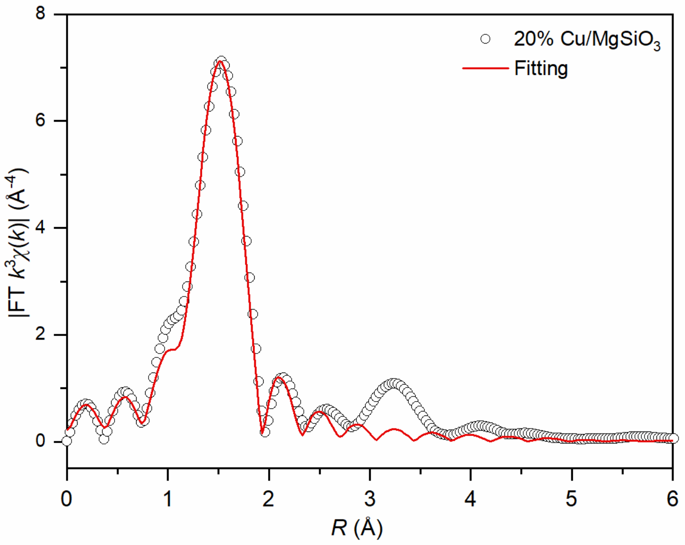

Figure S25. EXAFS fitting curve for 20% Cu/$MgSiO_3$. The corresponding k^3-weighted Fourier-transformed extended X-ray absorption fine structure (FT-EXAFS) spectra (Fig. S24b) and EXAFS curve fitting analysis (Fig. S25 and Table S5) further corroborate this result by indicating that the Cu–O bonds of the 20% Cu/$MgSiO_3$ catalyst have similar coordination environments with that of the $CuSiO_3$. Based on these results, it can be affirmed that the Cu elements exist in the forms of absorbed Cu species and $CuSiO_3$ [1].

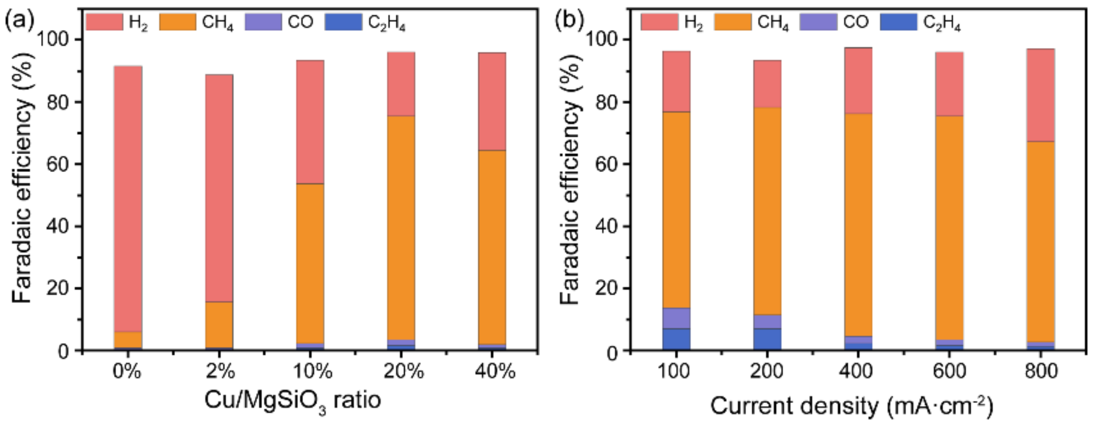

Figure S26. (a) The major CO_2RR product distribution of catalysts with different Cu/$MgSiO_3$ ratios at a current density of 600 mA/cm^2. (b) The Faradaic efficiency for CO_2RR products of 20% Cu/$MgSiO_3$ catalysts under different current densities.

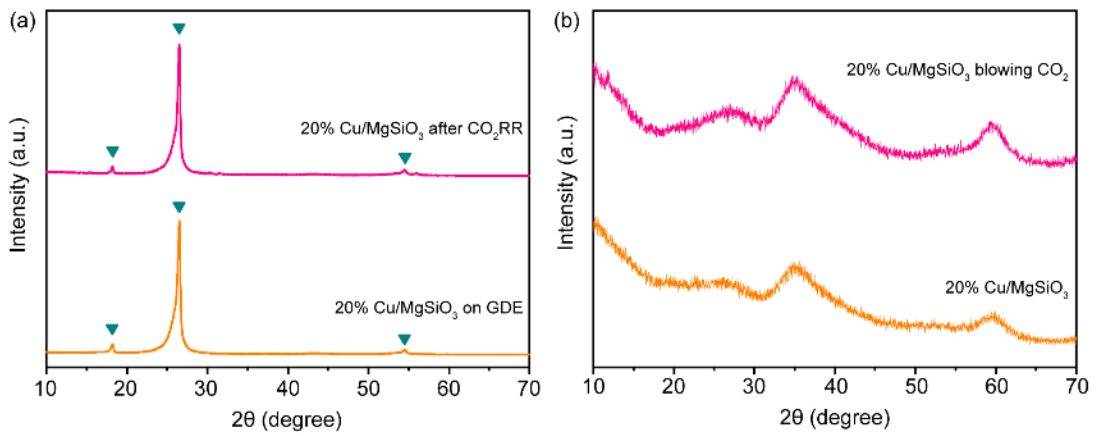

Figure S27. (a) Comparison of XRD patterns of 20% Cu/$MgSiO_3$ before and after CO_2RR. The XRD profiles of blank GDE are marked with inverted triangles. (b) Comparison of XRD patterns of 20% Cu/$MgSiO_3$ before and after blowing CO_2. No significant changes can be observed in the 20% Cu/$MgSiO_3$ after undergoing various reaction environments, suggesting its high stability.

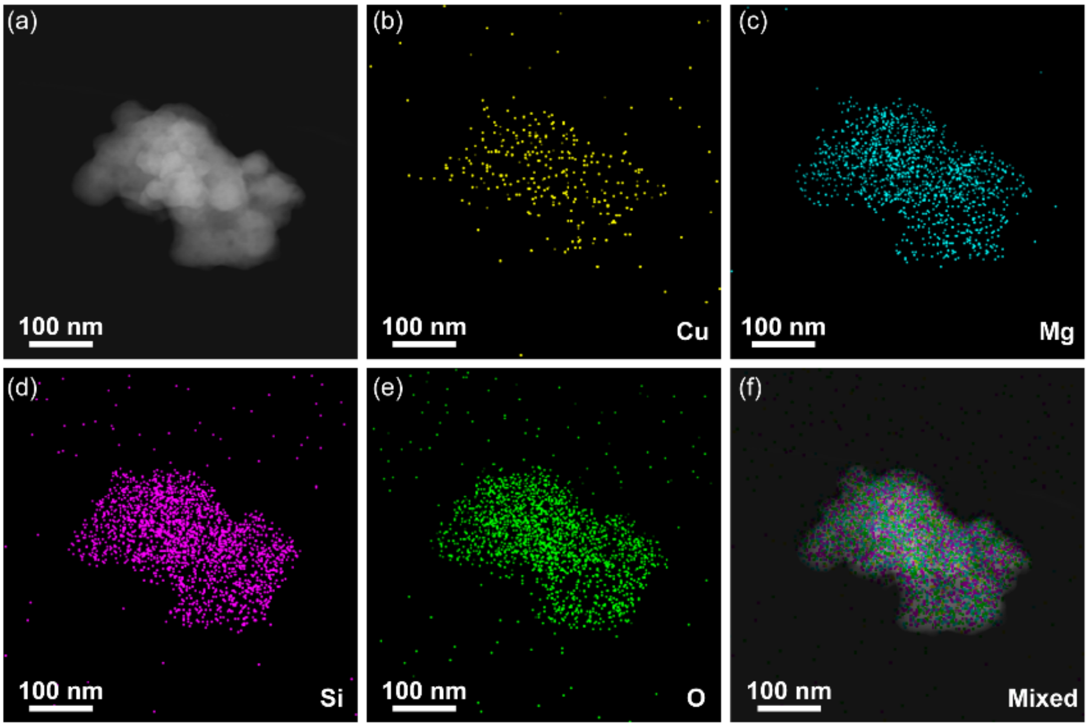

Figure S28. Elemental mapping images of 20% Cu/$MgSiO_3$ catalyst after electrochemical CO_2RR for Cu, Mg, Si and O elements. No obvious change can be observed between the elemental mapping images of 20% Cu/$MgSiO_3$ before and after the test, suggesting its high electrocatalytic stability.

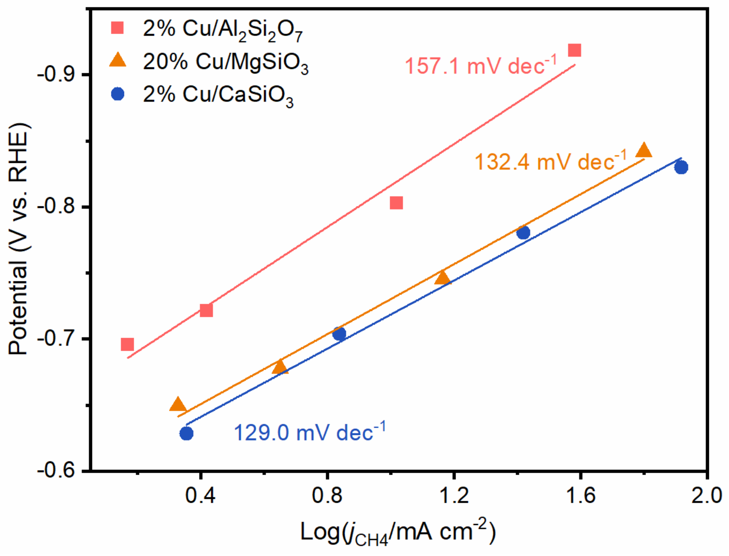

Figure S29. Tafel plots for CH_4 production of 2% Cu/$Al_2Si_2O_7$, 2% Cu/$CaSiO_3$ and 20% Cu/$MgSiO_3$. The Tafel slopes of 20% Cu/$MgSiO_3$ (132.4 mV dec^{-1}) and 2% Cu/$CaSiO_3$ (129.0 mV dec^{-1}) are determined to be lower than that of the 2% Cu/$Al_2Si_2O_7$ (157.1 mV dec^{-1}), manifesting their better reaction kinetic for electrochemical CO_2RR.

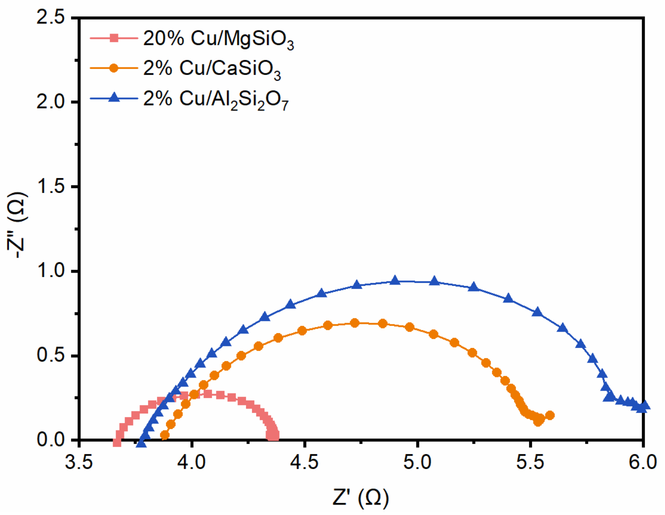

Figure S30. The Nyquist plots of 2% Cu/$Al_2Si_2O_7$, 2% Cu/$CaSiO_3$ and 20% Cu/$MgSiO_3$. The 20% Cu/$MgSiO_3$ demonstrates the smallest arc diameter among all the prepared samples, indicating its smallest charge transfer resistance and fastest electron transfer across surface and electrolyte. Such a superior electron transfer rate can allow more electrons to participate in the electrochemical CO_2RR, enabling the multi-electron reaction on the 20% Cu/$MgSiO_3$ toward CH_4 production.

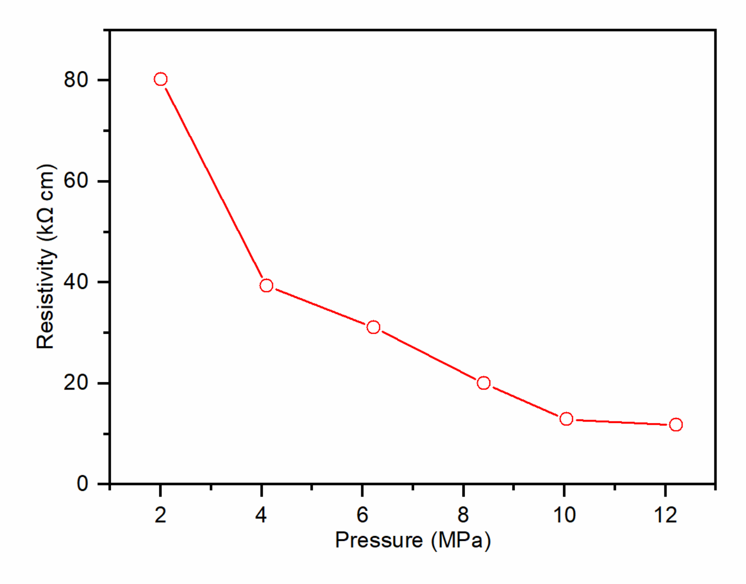

Figure S31. Bulk resistivity plot of hydrated $MgSiO_3$. Typically, the anhydrous $MgSiO_3$ demonstrates an insulator characteristic, whose electrical resistivity is over the detection limit of the bulk resistivity measurement. Interestingly, it behaves differently after hydration. In detail, $MgSiO_3$ can be hydrated during the reaction, forming rich hydroxyl groups on its surface, which can significantly enhance electrical conductivity [2,3]. Therefore, it demonstrates a conductor characteristic after hydration, showing an electrical resistivity down to 12 kΩ cm in the bulk resistivity measurement (Fig. S31). Such a conductor characteristic allows the efficient electrons transport from the $MgSiO_3$ to the Cu species during the electrocatalytic reaction.

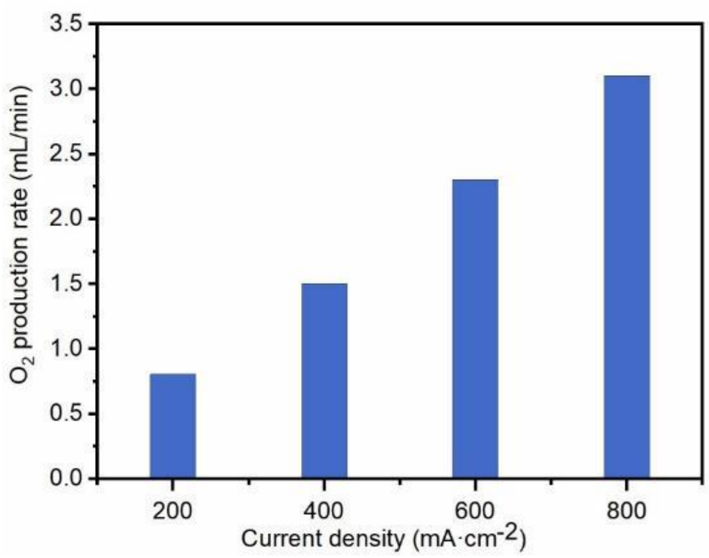

Figure S32. The production rates of O_2 when performing CO_2 electrolysis using 20% Cu/$MgSiO_3$ as the cathodic catalyst.

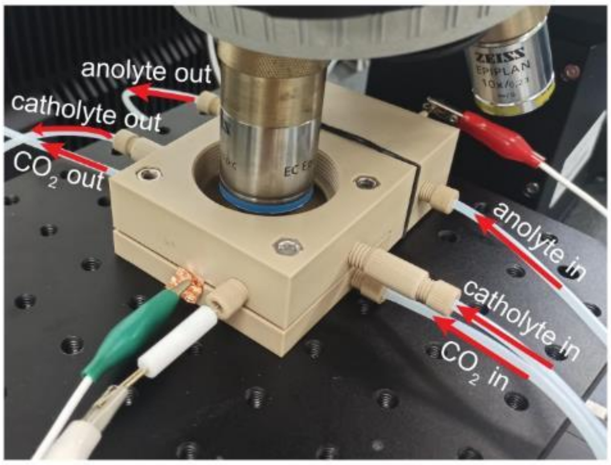

Figure S33. Photograph of the experimental setup for *in situ* Raman spectroscopy measurements.

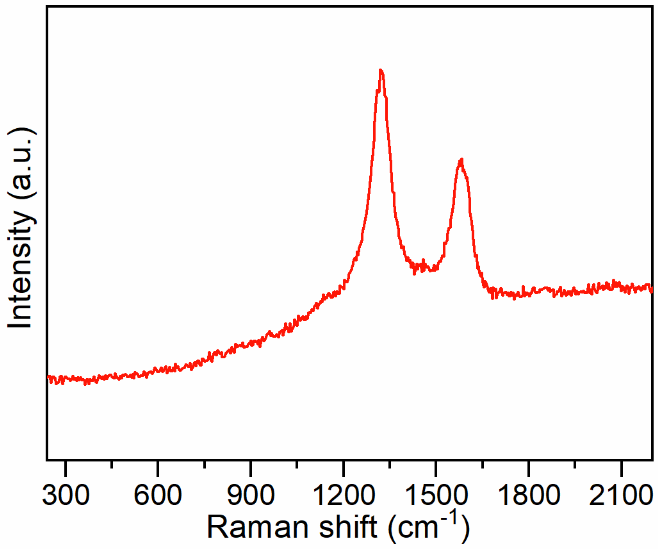

Figure S34. The Raman spectrum of the gas diffusion layer.

Table S1. The raw material fed in the catalyst preparation process and the actual Cu loading content on the catalysts.

Sample	The mass of solid	V_{water} (mL)	[a]V_{Cu} (mL)	[b]Actual Cu loading content	[b]Cu content after CO_2R
500% Cu/Augite-E	10 mg	/	5	0.63%	
0.2% Cu/$Al_2Si_2O_7$	100 mg	9.98	0.02	0.07%	
2% Cu/$Al_2Si_2O_7$	100 mg	9.8	0.2	0.17%	0.14%
20% Cu/$Al_2Si_2O_7$	100 mg	8	2	0.20%	
0.5% Cu/$CaSiO_3$	100 mg	9.95	0.05	0.15%	
2% Cu/$CaSiO_3$	100 mg	9.8	0.2	0.69%	0.57%
20% Cu/$CaSiO_3$	100 mg	8	2	7.24%	
10% Cu/$MgSiO_3$	100 mg	9	1	4.30%	
20% Cu/$MgSiO_3$	100 mg	8	2	6.89%	5.40%
40% Cu/$MgSiO_3$	100 mg	6	4	10.38%	

[a]V_{Cu} represents the adding volume of $CuCl_2 \cdot 2H_2O$ (10 mg/mL).

[b]Cu loading content of catalysts was determined by inductively coupled plasma optical emission spectrometry (ICP-OES).

Table S2. The Faradaic efficiencies for various products obtained via electrocatalytic CO_2 conversion over Cu/lunar soil at different current densities.

Current density (mA/cm^2)	FE_{HCOOH} (%)	FE_{CH_3COOH} (%)	$FE_{CH_3CH_2OH}$ (%)	FE_{H_2} (%)	FE_{CO} (%)	FE_{CH_4} (%)	$FE_{C_2H_4}$ (%)	FE_{Total} (%)
100	2.65	1.30	0.00	50.91	16.85	16.81	4.75	93.27
200	2.14	1.35	0.00	57.72	17.14	15.83	5.19	99.37
400	1.09	0.54	1.68	52.30	9.07	21.80	6.42	92.89
600	0.78	0.78	1.02	63.60	4.02	20.63	3.12	93.95

Table S3. The production rates of the gaseous products via electrocatalytic CO_2 conversion over 20% Cu/$MgSiO_3$ at a temperature of 298.15 K and pressure of 101.325 kPa.

Current density (mA/cm^2)	H_2 (mL/min)	CO (mL/min)	CH_4 (mL/min)	C_2H_4 (mL/min)
600	0.94	0.08	0.82	0.01
800	1.82	0.08	0.98	0.01

Table S4. Comparison of CH_4 Faradaic efficiencies for the recently reported Cu-based electrocatalysts.

Sample	Reactor	Electrolyte	Potential (vs. RHE)	j_{CH_4} (mA/cm^2)	FE_{CH_4}	Ref.
20% Cu/$MgSiO_3$	Flow cell	1 M KOH	−1.03	432.3	72.05%	This work
20% Cu/$MgSiO_3$	Flow cell	1 M KOH	−1.22	516.72	64.59%	This work
CeO_2 matrix with Cu^{2+}	Flow cell	1 M KOH	−0.82	135.6	67.8%	[4]
7% Au–Cu	Flow cell	1 M $KHCO_3$	−1.23	112	56%	[5]
NNU-33(H)	Flow cell	1 M KOH	−0.9	321.9	82%	[6]
Cu-DBC	Flow cell	1 M KOH	−0.9	162.4	80%	[7]
Cu-TDPP-NS	Flow cell	0.5 M PBS	−1.6	128.1	70%	[8]
$CuGaO_2$	Flow cell	1 M KOH	−2	717	71.7%	[9]
La_2CuO_4	Flow cell	1 M KOH	−1.40	117.0	56.3%	[10]
Cu/Al_2O_3	Flow cell	1 M KOH	−1.20	94.8	62%	[11]
CoO/Cu	Flow cell	1 M $KHCO_3$	−1.1	135	60%	[12]
Cu octahedra	Flow cell	1 M KOH	−0.91	53	53%	[13]
Sputtered Cu on PTFE	Flow cell	1 M $KHCO_3$	−1.0	108	48%	[14]
Carbon coated on Cu/Cu_2O core	Flow cell	1 M KOH	/	366.5	73.3%	[15]
Carbon coated on Cu/Cu_2O core	H-cell	0.1 M $KHCO_3$	−1.6	39	81%	[15]
Ag@Cu_2O	Flow cell	1 M KOH	−1.2	178	74%	[16]
CNP:CuPc	MEA	0.05 M $KHCO_3$	/	136	62%	[17]
Cu clusters/DRC	H-cell	0.1 M $KHCO_3$	−1.0	18	81.7%	[18]

Table S5. Fitting results of Cu K-edge EXAFS data.

Sample	Bond	[a]CN	[b]R (Å)	[c]σ^2 (Å^2)
$CuSiO_3$	Cu–O	4.03 ±0.15	1.94 ±0.01	0.00478
20% Cu/$MgSiO_3$	Cu–O	4.00 ±0.20	1.96 ±0.02	0.00498

[a]CN represents the coordination numbers; [b]R represents the bonding distance; [c]σ^2 represents the Debye-Waller factor.

References

1. Ravel B and Newville M. ATHENA, ARTEMIS, HEPHAESTUS: data analysis for X-ray absorption spectroscopy using ifeffit. *J Synchrotron Radiat* 2005. **12**: 537–41.
2. Nagao M, Kobayashi K and Jin Y *et al.* Ionic conductive and photocatalytic properties of cementitious materials: calcium silicate hydrate and calcium aluminoferrite. *J Mater Chem A* 2020; **8**: 15157–66.
3. Karato S. The role of hydrogen in the electrical conductivity of the upper mantle. *Nature* 1990; **347**: 272–3.
4. Zhou X, Shan J and Chen L *et al.* Stabilizing Cu^{2+} ions by solid solutions to promote CO_2 electroreduction to methane. *J Am Chem Soc* 2022; **144**: 2079–84.
5. Wang X, Ou PF and Wicks J *et al.* Gold-in-copper at low *CO coverage enables efficient electromethanation of CO_2. *Nat Commun* 2021; **12**: 3387.
6. Zhang L, Li XX and Lang ZL *et al.* Enhanced cuprophilic interactions in crystalline catalysts facilitate the highly selective electroreduction of CO_2 to CH_4. *J Am Chem Soc* 2021; **143**: 3808–16.
7. Zhang Y, Dong LZ and Li S *et al.* Coordination environment dependent selectivity of single-site-Cu enriched crystalline porous catalysts in CO_2 reduction to CH_4. *Nat Commun* 2021; **12**: 6390.
8. Wang YR, Liu M and Gao GK *et al.* Implanting numerous hydrogen-bonding networks in a Cu-porphyrin-based nanosheet to boost CH_4 selectivity in neutral-media CO_2 electroreduction. *Angew Chem Int Ed* 2021; **60**: 21952–8.
9. Peng C, Xu Z and Luo G *et al.* Highly-exposed single-interlayered Cu edges enable high-rate CO_2-to-CH_4 electrosynthesis. *Adv Energy Mater* 2022; **12**: 2200195.
10. Chen S, Su Y and Deng P *et al.* Highly selective carbon dioxide electroreduction on structure-evolved copper perovskite oxide toward methane production. *ACS Catal* 2020; **10**: 4640–6.
11. Chen S, Wang B and Zhu J *et al.* Lewis acid site-promoted single-atomic Cu catalyzes electrochemical CO_2 methanation. *Nano Lett* 2021; **21**: 7325–31.
12. Li Y, Xu A and Lum Y *et al.* Promoting CO_2 methanation via ligand-stabilized metal oxide clusters as hydrogen-donating motifs. *Nat Commun* 2020; **11**: 6190.
13. De Gregorio GL, Burdyny T and Loiudice A *et al.* Facet-dependent selectivity of Cu catalysts in electrochemical CO_2 reduction at commercially viable current densities. *ACS Catal* 2020; **10**: 4854–62.
14. Wang X, Xu A and Li F *et al.* Efficient methane electrosynthesis enabled by tuning local CO_2 availability. *J Am Chem Soc* 2020; **142**: 3525–31.
15. Zhang XY, Li WJ and Wu XF *et al.* Selective methane electrosynthesis enabled by a hydrophobic carbon coated copper core-shell architecture. *Energy Environ Sci* 2022; **15**: 234–43.
16. Xiong L, Zhang X and Chen L *et al.* Geometric modulation of local CO flux in Ag@Cu_2O nanoreactors for steering the CO_2RR pathway toward high-efficacy methane production. *Adv Mater* 2021; **33**: 2101741.
17. Xu Y, Li F and Xu A *et al.* Low coordination number copper catalysts for electrochemical CO_2 methanation in a membrane electrode assembly. *Nat Commun* 2021; **12**: 2932.

18. Hu Q, Han Z and Wang X *et al.* Facile synthesis of sub-nanometric copper clusters by double confinement enables selective reduction of carbon dioxide to methane. *Angew Chem Int Ed* 2020; **59**: 19054–9.
